# *Liolophura* species discrimination with geographical distribution patterns and their divergence and expansion history on the northwestern Pacific coast

**DOI:** 10.1038/s41598-021-96823-5

**Published:** 2021-09-02

**Authors:** Eun Hwa Choi, Mi Yeong Yeo, Gyeongmin Kim, Bia Park, Cho Rong Shin, Su Youn Baek, Ui Wook Hwang

**Affiliations:** 1 Department of Biology Education, Teachers College & Institute for Phylogenomics and Evolution, Kyungpook National University, Daegu, 41566 South Korea; 2 Institute for Korean Herb-Bio Convergence Promotion, Kyungpook National University, Daegu, 41566 South Korea; 3 School of Life Sciences, Graduate School, Kyungpook National University, Daegu, 41566 South Korea; 4 School of Industrial Technology Advances, Kyungpook National University, Daegu, 41566 South Korea

**Keywords:** Ecology, Evolution, Genetics, Zoology

## Abstract

The chiton *Liolophura japonica* (Lischke 1873) is distributed in intertidal areas of the northwestern Pacific. Using *COI* and *16S rRNA,* we found three genetic lineages, suggesting separation into three different species. Population genetic analyses, the two distinct *COI* barcoding gaps albeit one barcoding gap in the *16S rRNA,* and phylogenetic relationships with a congeneric species supported this finding. We described *L*. *koreana*, sp. nov. over ca. 33°24′ N (JJ), and *L*. *sinensis*, sp. nov. around ca. 27°02′–28°00′ N (ZJ). We confirmed that these can be morphologically distinguished by lateral and dorsal black spots on the tegmentum and the shape of spicules on the perinotum. We also discuss species divergence during the Plio-Pleistocene, demographic expansions following the last interglacial age in the Pleistocene, and augmentation of *COI* haplotype diversity during the Pleistocene. Our study sheds light on the potential for *COI* in examining marine invertebrate species discrimination and distribution in the northwestern Pacific.

## Introduction

Chitons (Polyplacophora, Neoloricata, and Chitonida) are marine mollusks of the class Polyplacophora that possess a dorsal shell, which is composed of eight separate calcium carbonate plates^1^. Nearly a thousand extant chiton species are distributed worldwide, and over 430 fossil species have been reported, stretching back ca. 300 million years, from the late Ordovician to the Early Periman age^1,2^; some have been dated as early as 500 million years old^3,4^. Since there are no significant differences in appearance between chiton fossils and extant species, they have been regarded as living fossils that have retained their past life history and ecological traits^5^. According to the classification by Sirenko^6^, modern chitons belong to two different orders of Lepidopleurida and Chitonida, the latter of which is subdivided into the suborders Chitonina and Acanthochitonina based on morphological characteristics. The genus *Liolophura* (family Chitonidae), belonging to the suborder Chitonina, contains only six species^2^: *L*. *gaimardi*, *L*. *hirtosa*, *L*. *rehderi*, and *L*. *arenosa* are distributed in the southern hemisphere (Australian continent), and only *L*. *japonica* and *L*. *tenuispinosa* are distributed in the northern hemisphere (Asian continent).

It is known that *L*. *japonica* is widely distributed throughout the intertidal coast of the northwestern Pacific, encompassing the Korean Peninsula, the Japanese Archipelago, and southern China. Okoshi and Hamaguchi^7^ found two different morphological types, A and B, of *L*. *japonica* in Japanese coastal regions, with supporting evidence of *COI* barcoding region sequence of an individual of each type. They suggested that the type A lacks black spots on the lateral areas of the tegmentum that normally appear in Onagawa Bay in the coastal area of northern Japan, whereas the type B appears in Nabeta Bay in the coastal area of southern Japan, and has distinct black spots. Despite their meaningful work, these authors used only a small number of individuals of *L*. *japonica* inhabiting the Japanese Archipelago in the northwestern Pacific, and employed partial *COI* sequencing of only a single individual from each type. Over the past decade, their population genetic and systematic studies have rarely been performed and geographical distribution patterns been scarcely studied. Recently, Wu et al.^8^ reported population genetic research of *L*. *japonica* based on *COI* and *16S rRNA* haplotypes of 125 individuals collected from the Zhejiang Province located at ca. 27°02′–28°00′ N in southern China. However, only samples from the Zhejiang Province were used, which are similar to type B in the appearance of black spots on the lateral areas of the tegmentum.

Here, we examine species discrimination probability, geographical distribution pattern, and demographical history of *L*. *japonica* in the northwestern Pacific based on 106 *COI* haplotypes from 469 individuals and 34 *16S rRNA* haplotypes from 425 individuals in total, with the addition of 342 new individuals collected directly from coastal areas of South Korea and Japan. Based on these results and those of previous studies^9,10^, we describe and discuss the species discrimination of *L*. *japonica* into three different species, including *L*. *koreana*, sp. nov., *L*. *japonica*, and *L*. *sinensis*, sp. nov., of which geographical distribution patterns, molecular divergence times, and demographical expansion history are analyzed and discussed.

## Results

### Sample collection of *Liolophura japonica* and the genetic diversity of *COI* barcoding region

**Figure 1:**
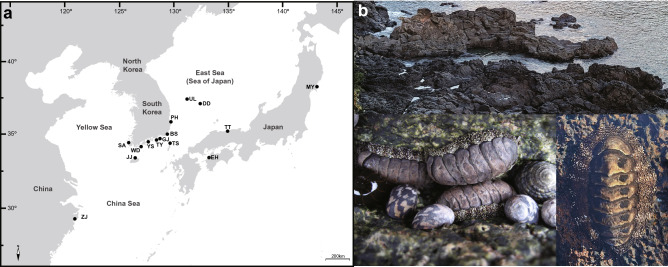
A map showing sampling localities and photos of a habitat landscape and wild samples of *Liolophura japonica* inhabiting coastal areas of the Korean Peninsula (*N* = 249), the Japanese Archipelago (*N* = 57), and southern China (*N* = 125) in the northwestern Pacific Ocean. **(a)** A map showing twelve direct sampling localities for *L*. *japonica* in coastal areas of the northwestern Pacific Ocean. The sampling localities of one southern Chinese (ZJ) and two Japanese (EH and MY) previously catalogued haplotype sequencing studies retrieved from NCBI are also depicted. Table S1 and S2 contain more accurate information on the populations and individuals. The basic map is from a free map providing site (https://d-maps.com), which is modified with Adobe Illustrator v.25.2. (https://www.adobe.com). **(b)** Photos of a habitat landscape and wild samples of *L*. *japonica*, taken from Seogwipo-si, Jeju Island, South Korea, photographed by Mi Young Yeo, Bia Park, and Cho Rong Shin. The photos were edited using Adobe Photoshop v.22.2 (https://www.adobe.com).

To examine genetic lineage divergence within *L*. *japonica* on the northwestern Pacific coast, we newly collected a total of 342 *L*. *japonica* samples from 12 sampling localities in the intertidal coasts of the Korean Peninsula and Japanese Archipelago (Fig. 1; Table S1). From the collected *L*. *japonica* samples, we amplified the *COI* barcoding region using PCR, and then sequenced the 635-bp PCR products. As a result, a total of 75 *COI* haplotypes based on *COI* sequences obtained from 342 individuals of *L*. *japonica* were detected via the present study (Table S2). In addition to this, we extracted 31 *COI* haplotypes based on *COI* sequences from 127 individuals of *L*. *japonica* (also known as *Acanthopleura japonica*) previously reported in the NCBI GenBank database, consisting of two Japanese and 29 Chinese *COI* haplotypes. Finally, we gathered 106 *COI* haplotypes from 469 *L*. *japonica* individuals collected in 15 localities of South Korea, Japan, and southern China (Tables S1, S2). The average haplotype (*h*) and nucleotide diversities (*π*) were 0.808 and 0.04936, respectively; the highest haplotype diversity was observed in Tsushima (TS; *h* = 0.963), and the highest nucleotide diversity was found in Wando (WD; *π* = 0.04581), located in the South Sea of the Korean Peninsula. As shown in Table S3, the population distribution pattern of *COI* haplotypes revealed that all collection sites had site-specific haplotype(s) except for Busan (BS), Wando (WD), Sinan (SA), and Jeju Island (JJ). The most abundant haplotype was A1, which was found in 170 (39.4%) out of the *COI* sequences obtained from 469 *L*. *japonica* individuals.

### Phylogenetic and population genetic analyses based on *COI*

**Figure 2:**
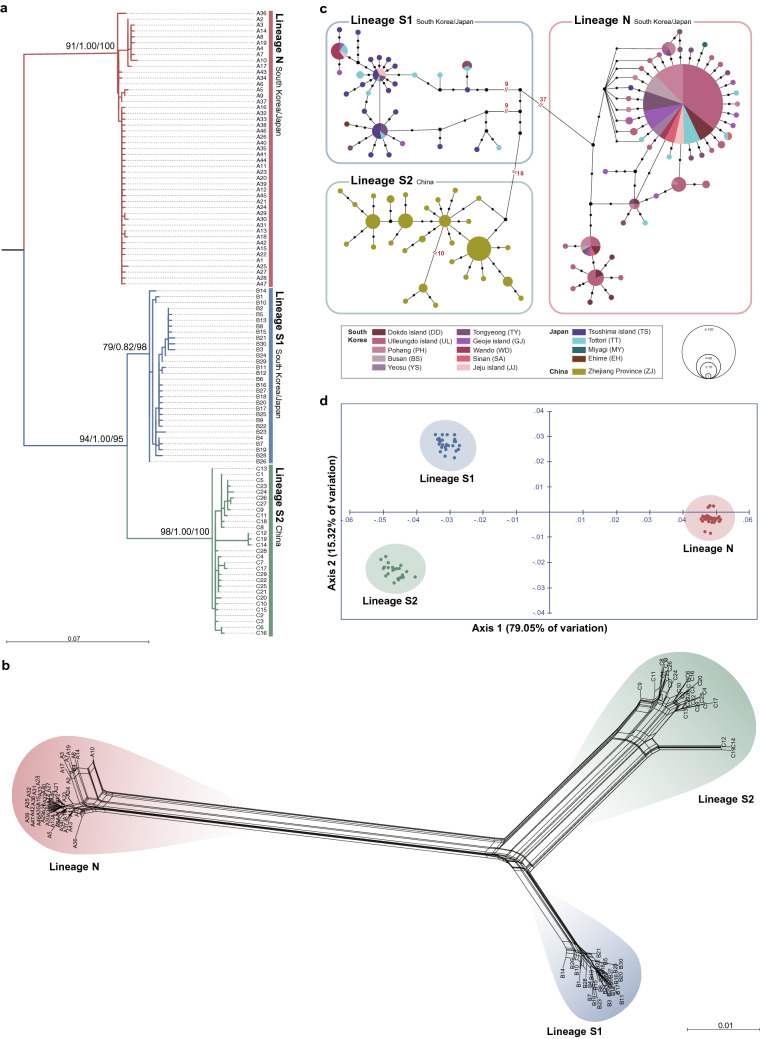
Phylogenetic, TCS network, and PCoA analyses based on 106 *COI* haplotypes from 469 individuals of *Liolophura japonica* inhabiting coastal areas of the northwestern Pacific Ocean, suggesting the existence of the three different genetic lineages: Lineage N, Lineage S1, and Lineage S2. **(a)** Maximum likelihood tree showing the three different genetic lineages for *L*. *japonica*: Lineage N members are most likely from the populations inhabiting a wide range of South Korea and Japan, Lineage S1 members from the populations inhabiting southern coastal areas of South Korea and Japan only, and Lineage S2 members from the southern Chinese population. As shown in Fig. S1, *Acanthopleura spinosa* was used as an outgroup. Numbers on branches indicate node confidence values: BP in ML, BPP in BI, and BP in NJ in order. **(b)** A phylogenetic network reconstructed using the neighbor net algorithm without an outgroup, showing three different genetic lineages for *L*. *japonica* inhabiting the northwestern Pacific coast: Lineages N, S1, and S2. The *COI* sequence alignment set used is shown in Data S1. Detailed information of the 106 *COI* haplotypes used in this phylogenetic analysis is summarized in Table S1 and S2. **(c)** An unrooted TCS network showing three distinct genetic clusters, corresponding to Lineages N, S1, and S2. Three different genetic groups correspond to the three genetic lineages shown in the phylogenetic tree (**a**), respectively. The haplotype frequency is displayed by the circle size. **(d)** A two-dimensional PCoA plot showing the three distinct genetic groups corresponding to Lineages N, S1, and S2 shown in the phylogenetic tree (**a**). The score on the first two axes (Axis 1 = 79.05% and Axis 2 = 15.32%) from the matrix of genetic distances estimated with the 106 *COI* haplotypes are indicated.

We constructed a nucleotide sequence alignment set with 106 *COI* haplotypes of *L*. *japonica* (Data S1), and identified 95 polymorphic sites (15.0%, Table S4) and 68 parsimoniously informative sites (10.7%). To elucidate phylogenetic relationships among the populations of *L*. *japonica*, we performed molecular phylogenetic analyses, including maximum likelihood (ML), Bayesian inference (BI), and neighbor-joining (NJ) analyses, based on these 106 *COI* haplotypes with the outgroup *Acanthopleura spinosa* (Fig. 2a, Figs. S1, S2). The resultant phylogenetic trees clearly revealed the existence of three distinct genetic lineages within the monophyletic group of *L*. *japonica* (100 BP in ML, 1.00 BPP in BI, and 100 BP in NJ): Lineage N (91 BP, 1.00 BPP, and 100 BP), Lineage S1 (79 BP, 0.82 BPP, and 98 BP), and Lineage S2 (98 BP, 1.00 BPP, and 100 BP). Among these three genetic lineages, Lineages S1 and S2 were grouped with high node confidence values (94 BP, 1.00 BPP, and 95 BP). We additionally conducted a phylogenetic network analysis using a neighbor net algorithm without an outgroup (Fig. 2b), which confirmed that these sequences were distinctly divided into three genetic lineages, in agreement with the topology of the rooted phylogenetic trees (Fig. 2a, Fig. S1).

Consistently, the TCS network analysis (Fig. 2c) and principal coordinate analysis (PCoA) (Fig. 2d) showed the existence of three distinguished genetic groups among *L*. *japonica*, in accordance with the phylogenetic analyses (Fig. 2a − b). The TCS network (Fig. 2c) revealed that Lineages S1 and S2 were separated by 18 mutation steps, which is far shorter than the distance between Lineages N and S1 (37 mutation steps) or between Lineages N and S2 (60 mutation steps), indicating that Lineages S1 and S2 have a close affinity and had only recently diverged from each other. The overwhelming dominancy of the A1 haplotype implies a recent and rapid population expansion of Lineage N. In addition to A1, it was found that haplotypes B2 for Lineage S1 and C21 for Lineage S2 were dominant. In the PCoA plot (Fig. 2d), the three genetic groups of *L*. *japonica* were also observed, as in the phylogenetic (Fig. 2a,b) and TCS network (Fig. 2c) analyses. Lineage N was distantly located from Lineages S1 and S2, while Lineages S1 and S2 were spatially much closer.

### Sample collection of *L. japonica* and the genetic diversity of *16S rRNA*

The 342 individuals of *L*. *japonica* from 12 localities in the intertidal coasts on the Korean Peninsula and Japanese Archipelago (Fig. 1) were subjected to PCR amplification of a partial region of *16S rRNA* (506 bp) (Tables S5, S6). Of these, only 299 samples were successfully amplified and sequenced. Based on 299 individual *16S rRNA* sequences, a total of 23 *16S rRNA* haplotypes of *L*. *japonica* were detected (Tables S5, S6). Combined with 11 haplotypes extracted from *16S rRNA* sequences of 125 *L*. *japonica* individuals known previously in southern China, we totaled 34 *16S rRNA* haplotypes from 425 *L*. *japonica* individuals in 13 collection localities. The average haplotype (*h*) and nucleotide (*π*) diversities were 0.702 and 0.02093, respectively; the highest haplotype diversity was found in Geojedo (GJ; *h* = 0.833), and the highest nucleotide diversity in Wando (WD; *π* = 0.02244), located in the South Sea of the Korean Peninsula. Overall, the average haplotype and nucleotide diversities of *16S rRNA* were lower than those of *COI* (Table S1). As shown in Table S7, the population distribution pattern of *16S rRNA* haplotypes revealed that most of the collection sites had site-specific haplotype(s), except for BS, GJ, WD, SA, JJ, and TT. The most abundant haplotype was RA1, which was found in 186 (48.1%) out of the *16S rRNA* sequences obtained from 425 *L*. *japonica* individuals.

### Phylogenetic and population genetic analyses based on *16S rRNA*

**Figure 3:**
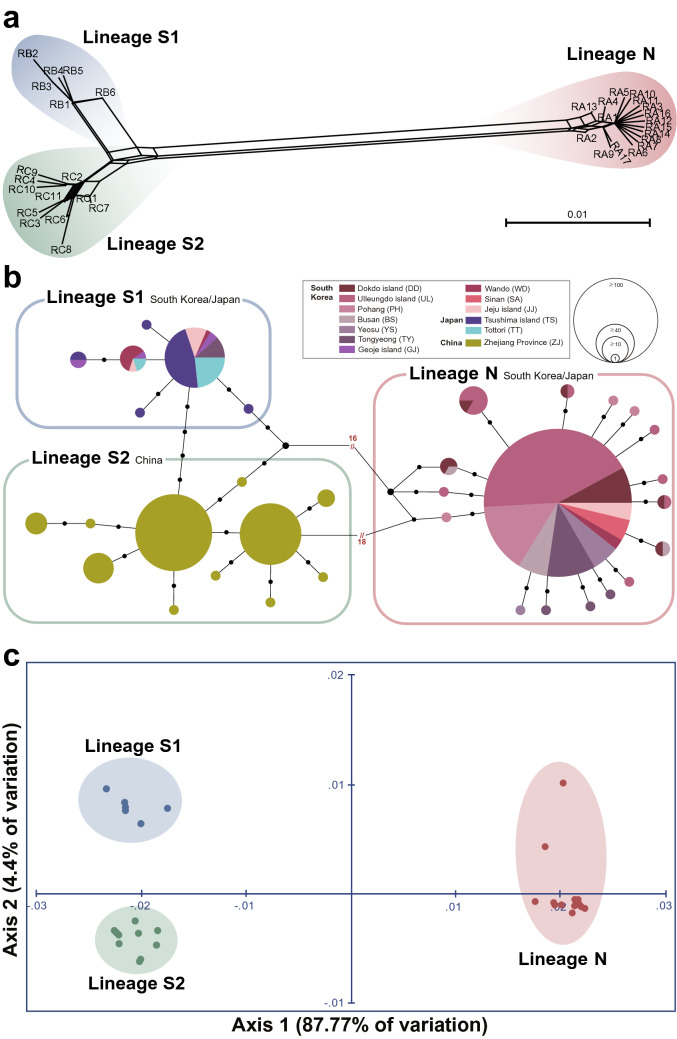
The results of phylogenetic and population genetic analyses based on 34 *16S rRNA* haplotypes from 425 individuals of *Liolophura japonica* inhabiting coastal areas of the northwestern Pacific Ocean. **(a)** Phylogenetic network reconstructed using the neighbor net algorithm, showing three different genetic lineages for *L*. *japonica*: Lineage N, Lineage S1, and Lineage S2. The *16S rRNA* sequence alignment set used is shown in Data S2. Detailed information of 34 *16S rRNA* haplotypes used in these analyses is summarized in Table S5 and S6. **(b)** An unrooted TCS network. There are distinctly observed three different genetic groups, corresponding to the three genetic lineages shown in the phylogenetic network (**a**). The haplotype frequency is displayed by the circle size. **(c)** A two-dimensional PCoA plot showing the three distinct genetic groups, corresponding to Lineage N, Lineage S1, and Lineage S2. The score on the first two axes (Axis 1 = 87.77% and Axis 2 = 4.4%) from the matrix of genetic distances estimated with the 34 *16S rRNA* haplotypes are indicated.

We constructed a nucleotide sequence alignment set with 34 *16S rRNA* haplotypes of *L*. *japonica* (Data S2), and identified 35 polymorphic sites (6.9%; Table S8) and 24 parsimoniously informative sites (4.7%). Phylogenetic analyses, including ML, BI, and NJ analyses, were conducted with the outgroup *Acanthopleura echinata* (Table S6). The resultant phylogenetic trees (Fig. S2) and unrooted phylogenetic network (Fig. 3a) consistently supported the three distinct genetic lineages of *L*. *japonica*, with the phylogenetic relationship between Lineages S1 and S2 being much closer than those inferred from the results of *COI* (Fig. 2a,b; Fig. S1). The TCS network (Fig. 3b) revealed that Lineages S1 and S2 were closely connected with only with 4–5 mutation steps between them, while Lineages N and S1 or Lineages N and S2 were remotely distanced by 18 mutation steps. Also, the overwhelming dominance of the RA1 haplotype implied a recent and rapid population expansion of Lineage N. In addition to RA1, haplotypes RB1 for Lineage S1 and RC1 and RC2 for Lineage S2 were dominant (Fig. 3b; Table S7). Consistent with this, in the PCoA plot (Fig. 3c), the three genetic groups of *L*. *japonica* were spatially separated. Lineage N was distantly located apart from Lineages S1 and S2, while Lineages S1 and S2 were spatially much closer.

### Examination of species discrimination of *L. japonica* based on *COI* and *16S rRNA*

**Figure 4:**
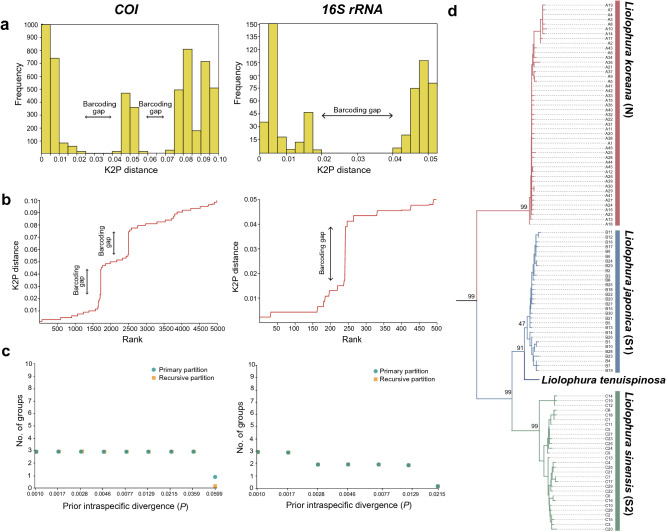
Distribution of pairwise genetic divergences, ranked pairwise difference, and automatic partition based on *COI* and *16S rRNA* haplotypes of *Liolophura japonica* and a COI-based NJ tree showing the phylogenetic relationship with a congeneric species *L. tenuispinosa*. **(a)** Distribution patterns of pairwise genetic divergences observed in *COI* and *16S rRNA* for *L*. *japonica*. The horizontal axis represents intervals of pairwise Kimura-2-parameter (K2P) genetic distance in percentage, and the vertical axis represents the number of individuals associated with each distance interval. **(b)** The results of ranked pairwise differences based on *COI* and *16S rRNA*, ranked by ordered value, which is similar to the distribution of pairwise genetic divergence in (**a**). The horizontal axis indicates a ranked ordered value based on K2P genetic distance, and the vertical axis represents the K2P genetic distance in percentage. **(c)** The results of automatic partition analyses based on *COI* and *16S rRNA*. The horizontal axis represents the prior maximum intraspecific divergence (*P*), and the vertical axis represents the number of groups inside the partitions (primary and recursive). **(d)** A *COI*-based NJ tree with *L. tenuispinosa*. Refer to Fig. S3 and Data S3.

Using the Automatic Barcode Gap Discovery (ABGD), we performed distribution of pairwise genetic divergences, ranked pairwise difference, and automatic partition analyses based on *COI* and *16S rRNA* of *L*. *japonica*, respectively (Fig. 4a–c), which confirmed that there were distinct barcoding gaps between intraspecific and interspecific variations, strongly supporting the possibility of species discrimination of *L*. *japonica*. the *COI*-based analysis yielded two different barcoding gaps, while the *16S rRNA*-based analysis revealed only a single barcoding gap (Fig. 4a–c). The results of automatic partition at each value of the prior intraspecific divergence (*P*) divided *L*. *japonica* into three groups by *COI* and two groups by *16S rRNA*, respectively (Fig. 4a–c). We also implemented two DNA taxonomy approaches to evaluate the possibility of species discrimination based on *COI*: the general mixed Yule coalescent (GMYC) approach (Fig. S3) and a Bayesian implementation of a Poisson Tree Processes model (bPTP) (Fig. S4). The results consistently and robustly supported the possibility that *L. japonica* can be divided into three different species, as shown in the results of ABDG (Fig. 4a–c).

The molecular variance analyses using analysis of molecular variance (AMOVA), based on *COI* and *16S rRNA*, were conducted to evaluate the degree of genetic differentiation among Lineages N, S1, and S2 (Tables S9, S10). According to the results, supposing that there are three genetic lineages (N, S1, and S2) or two genetic lineages (N and S1/S2), almost all variation in both cases is attributed to variation among groups (= among lineages), whereas variations within populations (within lineages) exhibit negative values in common. We confirmed that there was a high degree of genetic differentiation among Lineages N, S1, and S2, which supports the results of the *COI* barcoding gap analysis shown in Fig. 4a–c, although this was not statistically significant (*P* > 0.05; Table S9). When we assumed only two genetic groups, Lineages N and S1/S2, the genetic differentiation between the two groups was statistically significant (*P* < 0.001) in *COI* but not in *16S rRNA* (*P* > 0.05) (Tables S9 and S10). The discrepancy between the number of barcoding gaps inferred from *COI* and *16S rRNA* may have been affected by different gene evolutionary rates of the molecular markers^11^; nucleotide substitution rate of *16S rRNA* is known to be generally slower than that of *COI* (which is especially fast in the third codon positions: 105 out of 127 polymorphic sites). When an ML tree was constructed based on 22 polymorphic sites, which are found only in the first and second codon positions of *COI* that are much more conserved than the third codon position, the three genetic lineages were retained in the resultant tree (Fig. S5), but Lineage S2 was nested within Lineage S1, as in the trees inferred from *16S rRNA* (Fig. S2). Reflecting the powerful resolution of the *COI* barcoding marker well known from animals^12^ and the high degree of variation among the three genetic lineages (Fig. 4, Figs. S3, S4), we suggested that *L. japonica* could be categorized into three different species: *L. koreana*, Yeo and Hwang, sp. nov. for Lineage N, *L*. *japonica* for Lineage S1, and *L*. *sinensis* Choi, Park, and Hwang, sp. nov. for Lineage S2. To examine whether it is reasonable to give these a species-level taxonomic status, as shown in Fig. 4d, we reconstructed a *COI*-based NJ tree with one congeneric species *L. tenuispinosa*^13^, which was originally described as a subspecies-level taxon of *L. japonica*^14,15^ and was then revised as an independent species closely related to *L. japonica* by Saito & Yoshioka^16^ in 1993. The resultant tree (Fig. S6 and Data S3) showed that *L. tenuispinosa* forms a sister group with *L*. *japonica* (Lineage S1). This likely indicates that *L. koreana* and *L. sinensis* have taxonomic status as independent species.

### Morphological comparison and geographical distribution of the three *Liolophura* species

**Figure 5:**
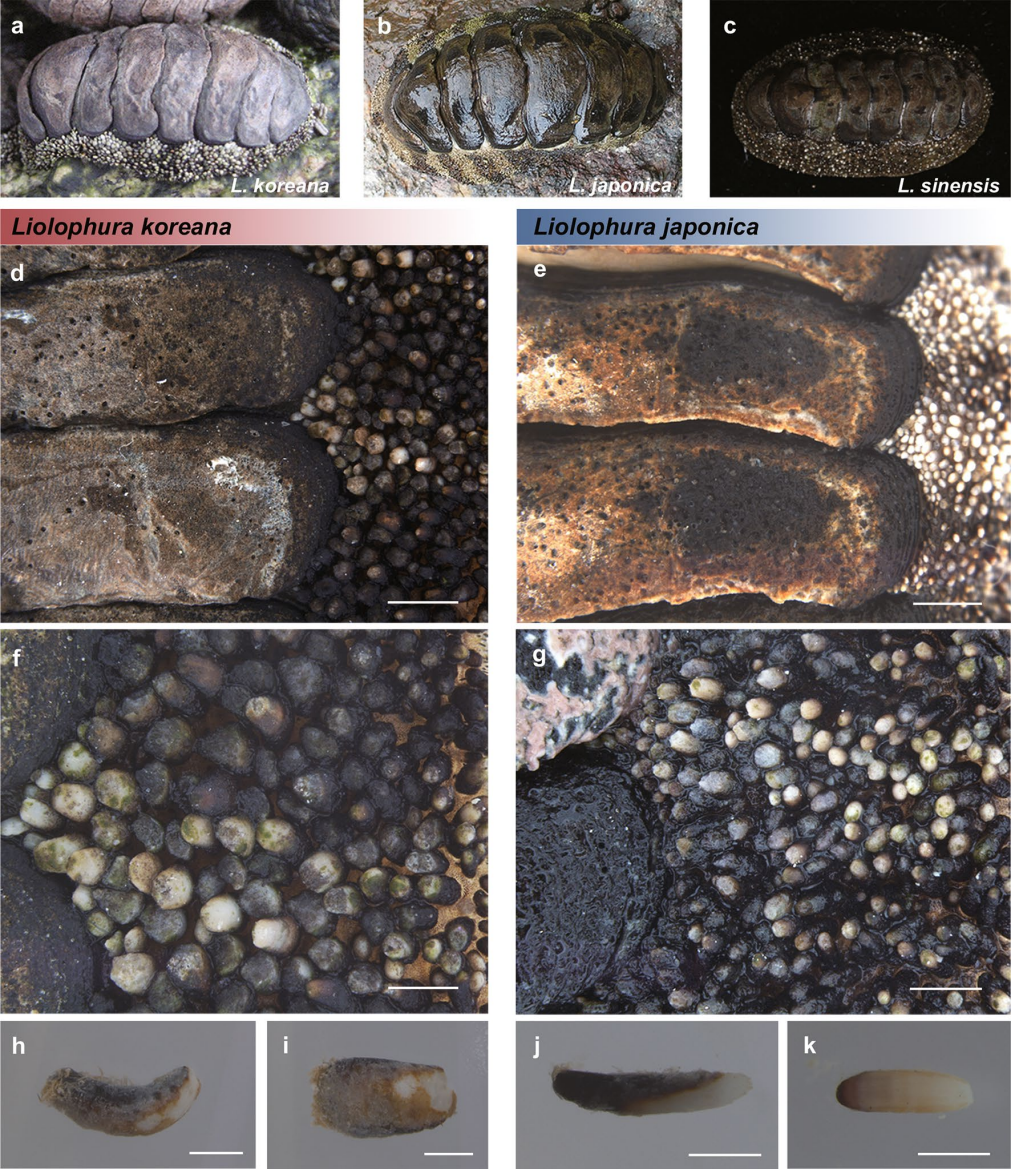
Morphological comparison of *Liolophura koreana*, sp. nov., *L. japonica*, and *L*. *sinensis*, sp. nov. **(a–c)** Photos of dorsal views of the individuals belonging to *L*. *koreana* (Lineage N), *L*. *japonica* (Lineage S1), and *L*. *sinensis* (Lineage S2) in order. **(d,e)** Morphological comparison of pleural and lateral black spots on valves III and IV of the tegmentum of *L*. *koreana* (d; holotype) and *L*. *japonica* (**e**). **(f,g)** Morphological comparison of spicules on the perinotum of *L*. *koreana* (**f**; holotype) and *L*. *japonica* (**g**). **(h–k)** Morphological comparison of the spicule of *L*. *koreana* (**h**,**i**; paratype) and *L*. *japonica* (**j**,**k**) in lateral and dorsal views. The scale bar marks 2.0 mm (**d**,**e**), 1.0 mm (**f**,**g**), and 0.5 mm (**h**–**k**). The photos were edited using Adobe Photoshop v.22.2 (https://www.adobe.com).

**Figure 6:**
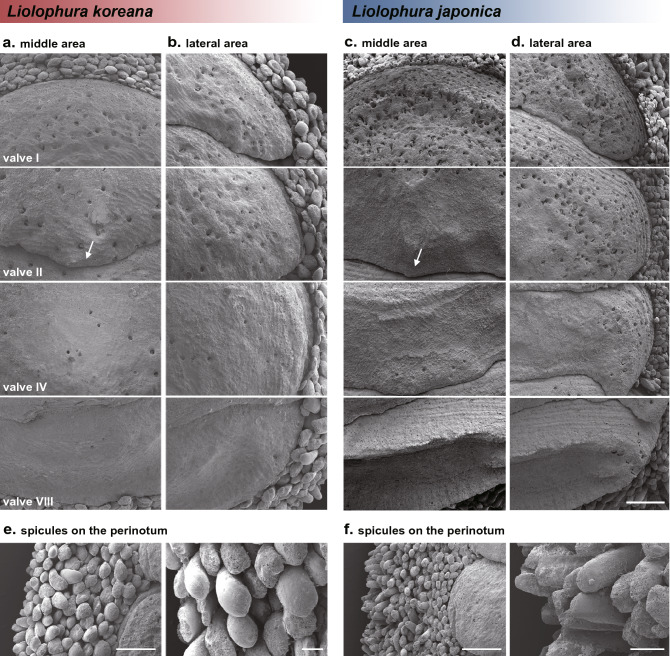
Microstructural comparison of *Liolophura koreana*, sp. nov. and *L. japonica* using field emission scanning electron microscopy (FE-SEM). **(a,b)** Middle and lateral areas on the tegmentum of the holotype of *L*. *koreana*. **(c,d)** Middle and lateral areas on the tegmentum of *L*. *japonica*. Arrows indicate that morphological difference of the posterior valve margin of the valve II between two species. The scale bar marks 1.0 mm. **(e,f)** The occurrence frequency, and shape and structure differences of the spicules on the perinotum between the holotype of *L*. *koreana* (**e**) and *L*. *japonica* (**f**). The scale bar marks 1.0 mm and 0.2 mm, respectively. The photos were edited using Adobe Photoshop v.22.2 (https://www.adobe.com).

We compared morphological characteristics among *Liolophura koreana*, sp. nov. (Lineage N), *L*. *japonica* (Lineage S1), and *L*. *sinensis*, sp. nov. (Lineage S2). Their morphological appearances are shown in Fig. 5a–c, which indicated that black spots on the tegmentum (Fig. 5d–e) and shapes of spicules on the perinotum (Figs. 5f–k, 6e–f) represent key morphological characteristics to distinguish them from each other. Although black dots in pleural areas, which are between the middle and lateral areas of the tegmentum on valves II–VII (or VIII), are commonly shared in all three lineages (Fig. 5a–c), other black spots on the valves exhibit a high degree of variation in morphology (Fig. 5a–c, Fig. S7). Herein, we described a new species of genus *Liolophura*, that is, *L*. *koreana* Yeo and Hwang from South Korea and Japan, with detailed descriptions of morphological characteristics observed by light microscopy (M205, Leica Camera AG, Germany) and FE-SEM (SU8220, Hitachi, Japan). In addition, we suggested the divergence of a new species, *L*. *sinensis* Choi, Park, and Hwang from southern China, with simple remarks based on distinct genetic difference (mainly *COI* barcoding gaps), with possible unique morphological characteristics as follows.

#### *Liolophura koreana* Yeo and Hwang, sp. nov. (Figs. 5, 6; Figs. S7, S8)

(urn:lsid:zoobank.org:act:4418355E-F55C-44FA-B4CE-585589FDCD23).

#### Type specimens examined

[Holotype] SOUTH KOREA: 1 specimen, Jeju-do, Seogwipo-si, Seongsan-eup, Seopjikoji, 3.XI.2020, UW Hwang, B Park & CR Shin (LEGOM040501); [Paratypes] SOUTH KOREA: 1 specimen, Gyeongsangbuk-do, Pohang-si, Guryongpo-eup, Janggil-ri, 27.VII.2008, UW Hwang (LEGOM040502); 3 specimens, Gyeongsangbuk-do, Ulleung-gun, Seo-myeon, Namyang-ri, Ulleungdo Island, Namtong tunnel, 12.VI.2007, UW Hwang (LEGOM040503–0505); 1 specimen, Gyeongsangbuk-do, Ulleung-gun, Namyang-ri, Ulleungdo Island, Namyang tunnel, 5.X.2007, UW Hwang (LEGOM040506); 2 specimens, Gyeongsangnam-do, Geoje-si, Nambu-myeon, Dapo-ri, 28.IV.2009, MY Yeo (LEGOM040507,0508); 4 specimens, same data as the holotype (LEGOM040509–0512); 2 specimens, Jeollanam-do, Yeosu-si, Hwajeong-myeon, Sado-ri, Sado Island, 8.IV.2008, MY Yeo (LEGOM040513,0514); 4 specimens, same data as the holotype (LEGOM040515–0518); JAPAN: 6 specimens, Tottori Prefecture, Hakuto, 24.V.2009, UW Hwang (LEGOM040519–0524); 1 specimen, Tottori Prefecture, Iwato, 25.V.2009, UW Hwang (LEGOM040525).

#### Description

Body small-sized and broad oval- to oval-shaped (Fig. 5a; Fig. S7); length 3.9 (1.9–12.3) mm and width 2.4 (1.2–7.1) mm. Tegmentum entirely brown (dark brown or black entirely, or each valve with black line anteriorly or white line laterally), with black dots on the pleural areas of valves II–VII (or VIII) (Fig. 5a,d, Fig. S7); articulamentum entirely black (dark brown); whitish and blackish spicules on the perinotum scattered irregularly, sometimes forming a band besides each valve (Fig. 5a, Fig. S7). Surface of the tegmentum in middle and lateral areas as in Fig. 6a,b and Fig. S8; posterior margin of the head valve nearly straight; dorsal shape of intermediate valves round-backed and side slopes slightly convex; the posterior valve margin with a distinct central apex, its shape subtriangular to triangular (rounded or linear), particularly valve II, mainly with a strong projection (Fig. 6a). Perinotum covered with large, solid, slightly curved, and obtusely pointed spicules (rarely with smooth and radial ribbed spicules apically), its density relatively lower than that of *L*. *japonica* (Figs. 5f,h,i, 6e).

#### Distribution

**Figure 7:**
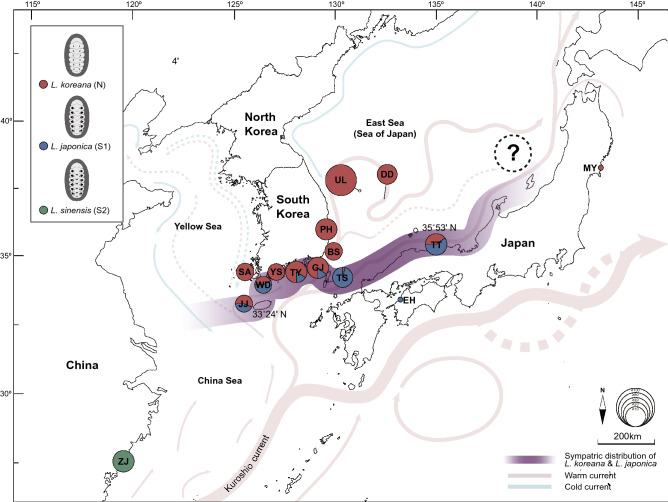
Geographical distribution of *Liolophura koreana*, sp. nov., *L. japonica*, and *L*. *sinensis*, sp. nov. inhabiting coastal areas of the northwestern Pacific Ocean. A *COI*-based map showing geographical distribution of *L*. *koreana*, *L*. *japonica*, and *L*. *sinensis* on the northwestern Pacific coast. *L*. *koreana* are found in a wide range of South Korea and Japan above ca. 33°24′ N (JJ), *L*. *japonica* in mainly southern coastal areas of South Korea and Japan below ca. 35°53′ N (TT), and *L*. *sinensis* in ZJ of southern China around ca. 27°02′ N–28°00′ N. The sympatric distribution of *L*. *koreana* and *L*. *japonica* is shown between 33°24′ and 35°53′ N. Table S1–S3 contain the full names of localities and detailed haplotype information. The question mark indicates that collection of *Liolophura* samples from such coastal areas in Japan is required to clarify distribution patterns of *L. koreana* and *L. japonica* in the East Sea (= Sea of Japan). The basic map was obtained from a free map-providing site (https://d-maps.com), which was modified using Adobe Illustrator v.25.2. (https://www.adobe.com).

South Korea, Japan; avobe 33°24′ N (Seogwipo, JJ) in South Korea and TT and MY in Japan (Fig. 7).

#### Habitat

This new species appears to be attached to rocks in coastal areas with strong waves, or a calm inner shore in the northwestern Pacific Ocean (Fig. S9).

#### Etymology

The species is named per the locality of the new species.

#### Remarks

We found that *Liolophura koreana*, sp. nov. has no black spots on lateral areas of the tegmentum (Fig. 5d), and large, slightly curved, and obtusely pointed spicules on the perinotum compared to those of *L*. *japonica* (Figs. 5f,h,i, 6e). On the other hand, *L*. *japonica* from southern South Korea and southern Japan has two black spots on the lateral areas of valves II–VII (or VIII) (Fig. 5e), and small, almost straight, and cylindrical spicules compared to those of *L*. *koreana* (Figs. 5g,j,k, 6f).

As shown in Fig. 7, *L*. *koreana* (Lineage N) was observed in all the South Korean and Japanese populations examined here, except for the EH population in Japan (refer to Tables S1, S2), which were found from JJ at 33°24′ N to MY at 38°32′ N. On the other hand, *L. japonica* (Lineage S1) was found from the southern coastal areas of South Korea and Japan, which were found only between JJ at 33°24′ N and TT at 35°53′ N. Interestingly, we found only *L*. *koreana* north from the latitude of 35°10′ N (BS) such as UL/DD (37°24′ N) and PH (36°02′ N) in South Korea. In Japan, there was found only *L*. *koreana* at MY (38°32′ N) too, but it remains to be explored to clarify its distribution range in Japan with much more sample collections covering northern Japanese coastal areas through further study. It was also confirmed that *L*. *koreana* and *L*. *japonica* show a sympatric distribution pattern between JJ at 33°24′ N and TT at 35°53′ N on the southern coastal area of South Korea and Japan.

#### *Liolophura sinensis* Choi, Park, and Hwang, sp. nov. (Fig. 5c).

(urn:lsid:zoobank.org:act:72DF7E75-1853-4F23-AC12-3AB8CD054187).

#### Type specimens examined

[Holotype] CHINA: 1 specimen, Zhejiang Province, Dongtou Island, 27°49′57.44″ N, 121°10′19.13″ E, 2017; [Paratypes] CHINA: 1 specimen, Beiji Island, 27°37′08.82″ N, 121°11′47.82″ E, 2017; 1 specimen, Beilongshan Island, 27°40′08.56″ N, 121°58′51.56″ E, 2017; 1 specimen, Chaishi Island, 27°25′40.36″ N, 121°04′54.45″ E, 2017; 1 specimen, Daleishan Island, 27°29′39.48″ N, 121°05′24.50″ E, 2017; 1 specimen, Daqu Island, 27°47′29.92″ N, 121°05′23.97″ E, 2017; 1 specimen, Dazhushi Island, 27°49′12.87″ N, 121°12′48.74″ E, 2017; 1 specimen, Dongce Island, 27°45′32.04″ N, 121°09′01.38″ E, 2017; 1 specimen, Dongxingzai Island, 27°02′40.36″ N, 121°02′47.98″ E, 2017; 1 specimen, Houjishan Island, 27°28′26.96″ N, 121°07′40.71″ E, 2017; 1 specimen, Luxi Island, 27°59′33.43″ N, 121°12′50.70″ E, 2017; 1 specimen, Nanji Island, 27°27′30.57″ N, 121°03′06.28″ E, 2017; 1 specimen, Nanpanshan Island, 28°00′15.29″ N, 121°15′33.62″ E, 2017.

#### Distribution

Southern China; Zhejiang Province around ca. 27°02′–28°00′ N (Fig. 7).

#### Habitat

This new species appears to be attached to rocks in coastal areas of the northwestern Pacific Ocean.

#### Etymology

The species is named after its locality.

#### Remarks

*L. sinensis*, sp. nov. was examined and established mainly by molecular data such as the *COI* barcoding gap (Fig. 4a–c) presented in this study and photos provided from Prof. Yong-Pu Zhang (Wenzhou University, Zhejiang Province, China) without any direct real sample observation. The morphology of this new species is very similar to that of a previously known species, *L*. *japonica*. *L*. *sinensis* from southern China has black spots on the lateral areas of valves II–VII similar to *L*. *japonica*, but black bowtie-shaped spots anterior to valves II–VII (Fig. 5c) are a unique characteristic for *L*. *sinensis*. *L*. *sinensis* (Lineage S2) was found in Zhejiang Province (ZJ) in southern China, around ca. 27°02′–28°00′ N (Fig. 7; Tables S1, S5).

### Demographic history and divergence time estimation analyses

**Figure 8:**
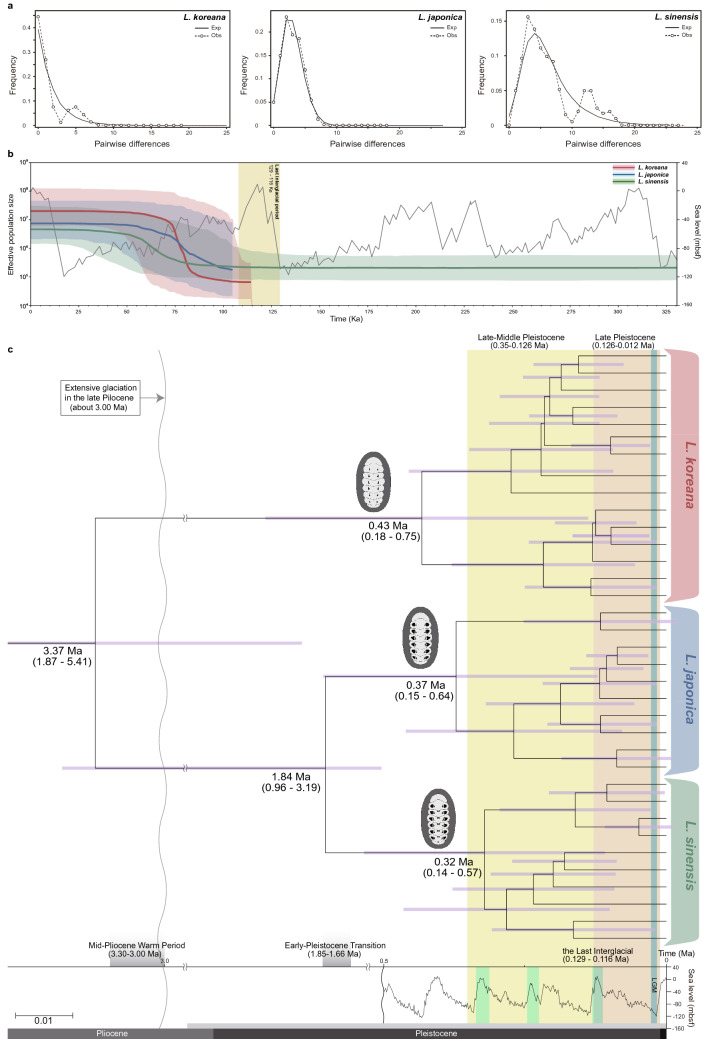
The results of mismatch distribution analyses (MDA), Bayesian skyline plots (BSPs), and molecular clock analysis performed with *COI* haplotypes for *Liolophura koreana*, sp. nov., *L. japonica*, and *L*. *sinensis*, sp. nov. **(a)** MDA plots resulting in a unimodal curve for *L*. *koreana*, *L*. *japonica*, and *L*. *sinensis*. Dotted lines indicate the observed distribution of mismatches, and solid lines represent the expected distribution under a demographic expansion model. **(b)** BSP results showing the demographic history of population expansions of *L*. *koreana*, *L*. *japonica*, and *L*. *sinensis*. The graph in gray depicts sea level changes during the last 330 Ka. **(c)** Time-calibrated Bayesian tree reconstructed using BEAST with the inference of ancestral areas under the Bayesian binary MCMC (BBM) model implemented in RASP ver 3.2. Ancestral areas were hypothesized based on the distribution range of the fossil records of *Mopalia* and the contemporary distribution of *L*. *koreana*, *L*. *japonica*, and *L*. *sinensis*. LGM indicates the last glacial maximum (0.026–0.019 Ma; blue vertical bar) and three interglacial periods are indicated by light green boxes during the late-middle and late Pleistocene. The pictures were edited using Adobe Illustrator v.25.2. (https://www.adobe.com).

Mismatch distribution analyses (MDA) based on *COI* were performed for *L*. *koreana*, *L*. *japonica*, and *L*. *sinensis*, respectively. The MDA results (Fig. 8a) showed a unimodal curve for each of the three lineages. In addition, when neutrality tests were performed with *COI* and *16S rRNA* (Table S11), all three showed statistically significant negative values in both Tajima’s *D* and Fu’s *F*s, except for the Tajima’s *D* values in *COI* (*L*. *sinensis*) and *16S rRNA* (*L. japonensis* and *L. sinensis*), implying that these had experienced population expansions. Bayesian skyline plot (BSP) analyses with *COI* (Fig. 8b) were performed to examine the fluctuation patterns in effective population sizes for *L*. *koreana*, *L*. *japonica*, and *L*. *sinensis*, respectively. The effective population sizes of *L*. *japonica* and *L*. *sinensis* had gradually grown between ca. 100 Ka and ca. 50 Ka, while those of *L*. *japonica* had grown between ca. 80–50 Ka, *L*. *sinensis* had grown between 100–60 Ka, and that of *L*. *koreana* had begun to rapidly expand ca. 85 Ka and ceased ca. 75 Ka. This indicated that population expansion had occurred more dramatic in *L*. *koreana* than in *L*. *japonica* and *L*. *sinensis*, following the last interglacial age, called the Eemian (129–116 Ka). As shown in Fig. 8c and Fig. S10, according to the molecular clock analysis by the BEAST program, it was estimated that *L*. *japonica* and *L*. *koreana* shared their most recent common ancestor about 3.37 Ma, around the mid-Pliocene warm period (3.30–3.00 Ma), before the extensive glaciation in the late Pliocene (ca. 3.00 mya). *L*. *japonica* and *L*. *sinensis* likely diverged around 1.84 Ma, around the beginning stage of the Early Pleistocene Transition (EPT; 1.85–1.66 mya). The augmentation of haplotype diversity in *L*. *japonica*, *L*. *sinensis*, and *L*. *koreana* might have intensified in the interglacial stages during the late-middle (0.35–0.126 Ma) and late Pleistocene (0.126–0.012 Ma), before the last glacial maximum (LGM: 0.026–0.019 Ma).

## Discussion

Three genetic lineages observed in *COI* and *16S rRNA* (Figs. 2, 3, 4; Figs. S1, S2) and morphological differences in lateral and dorsal black spots on the tegmentum and spicules on the perinotum (Figs. 5, 6), with the existence of two *COI* barcoding gaps and phylogenetic relationships with a congeneric species *L. tenuispinosa* (Figs. 4, S3, S4, S6), strongly support the independent species status of *L*. *koreana*, *L*. *japonica*, and *L. sinensis*.

Intriguingly, we also found that there are ecologically subtle differences in the microhabitats of *L*. *koreana* and *L*. *japonica*: *L*. *japonica* appears to prefer rock surfaces found only inside the shoreline, where there are few waves, whereas *L*. *koreana* appears to be attached onto rock surfaces regardless of whether it is in an area with strong ocean waves or a calm inner shore (Fig. S9). On the eastern coastline of the Korean Peninsula (PH) and volcanic islands in East Sea (UL and DD), rocks may be directly exposed to incoming strong ocean waves without any blocking or buffering due to simple coastline. In these sites, only *L*. *koreana* were found. On the contrary, the southern coastline of the Korean Peninsula (TY and GJ) and the islands located in South Sea of the Korean Peninsula (JJ and TS) have topographically complex coastline since it is ria coast, possibly providing various types of habitat conditions (i.e., strong sea waves can be weaker and seawater can be warmer). In these areas, both *L*. *koreana* and *L*. *japonica* may be found. The difference in geographic distribution of the two *Liolophura* species can be interpreted, at least in part, based on these physical differences in shorelines of northwestern Pacific Ocean. In addition, the southern coasts of the Korean Peninsula and the Japanese Archipelago are constantly under the direct influence of these warm currents (Fig. 7). In contrast, the seawater temperature on the east coast of the Korean Peninsula does not rise as high as the south ria coast, due to the influence of the cold Liman current that flows southward from Vladivostok, Russia, even though some of these warm currents travel northward through the East Sea (Japanese Sea) between the Korean Peninsula and the Japanese Archipelago. The difference in the seawater temperature may be one of the reasons why *L*. *japonica* is not observed on the east coast of the Korean Peninsula.

Recently, some studies have reported the delimited distribution of northern and southern types of marine invertebrates inhabiting coastal areas of the northwestern Pacific Ocean^17,18^. For instance, Xu et al.^18^ reported that the chiton *Acanthochitona rubrolineata* (Lischke, 1873) can be divided into the northern type found north of Lianyungang (LYG; ca. 34°59′ N) and the southern type found south of Zhousha (ZS; ca. 29°98′ N) on the East Chinese coast based on *COI*, *16S rRNA*, and *28S rRNA* sequences. In addition, Cheng and Sha^17^ reported cryptic diversity in the Japanese mantis shrimp *Oratosquilla oratoria* (De Haan, 1844) based on *COI* and ITS. According to their results, in the East Chinese coast, *O*. *oratoria* is divided into the northern type found north of Dafeng (DF; ca. 33°20′ N), and the southern type found south of Sheyang (SY; ca. 33°75′ N), exhibiting a sympatric distribution zone. On the Japanese coast, this species is divided into the northern type found north of the Araike Sea (AS; ca. 32°98′ N), and the southern type found south of Aomori (AO; ca. 40°82′ N), exhibiting a sympatric distribution zone. These recent studies, including our work, show that marine invertebrates exhibit distinct geographical distribution patterns (i.e. northern and southern types), which can be used for monitoring marine invertebrates whose migration north is fostered by global warming^19^. Further exploration of the detailed distribution patterns of the *Liolophura* species in the coastal areas of the northwestern Pacific uncovered in this study is required, with additional information on how long and how far the planktonic embryos and ciliated trochophore larvae float in the sea^20–25^.

Presumably, population expansions of the *Liolophura* species (Fig. 8b) might arise as a result of the last interglacial stage (129–116 Ka), called the Eemian interglacial period, named after the Eem River in the Netherlands, which was the last time when sea level was as high as or even higher than present-day sea level, and a time when the earth was slightly warmer than the present^26^. In addition, the molecular clock analysis (Figs. 8c, S10) estimated that *L*. *japonica* and *L*. *koreana* had diverged around the mid-Pliocene warm period^27^. During this period, the global average temperature was 2–3 °C higher^28^ and global sea level was 25 m higher^29^ than those observed today, transiently just before the onset of extensive glaciation over Greenland in the late Pliocene, approximately 3 Ma^30^. Since diversification of *L*. *japonica* and *L*. *sinensis* occurred during the beginning stage of the EPT (1.85–1.66 Ma), they passed through the Mid-Late and late Pleistocene periods (0.350–0.012 Ma) when sea levels dramatically and repeatedly fluctuated between glacial and interglacial stages. Considering possible errors in divergence time estimates, species diversification of *L. koreana* (northern lineage) and *L. japonica* (southern lineage) may have occurred during the Pliocene to the mid-Pleistocene (5.41–1.87 Ma; Fig. 8c) period. This approximation corresponds to the historical isolation of the East Sea (Sea of Japan) from the South and East China Seas, which was caused by a decline in the sea level during glacial cycles. Contemporary distributions of *L. koreana* (northern lineage) and *L. japonica* (southern lineage) are likely affected by two different surface water temperature zones in the northwestern Pacific^31^. In particular, after divergence, *L. koreana* and *L. japonica* may have adapted to the two aforementioned different surface water temperature zones. Subsequent species delineation of *L. sinensis* may have then occurred from the southern lineage (*L. japonica*) during the Pliocene to the late Pleistocene (3.19–0.96 Ma) period. Taken all together, *L*. *koreana*, *L*. *japonica*, and *L. sinensis* can be robustly separated with strong multi-pronged phylogenetic, population genetic, demographic, morphological, and ecological evidences. If several more samples are obtained from a wider range of coastal areas of the northwestern Pacific Ocean (particularly southern China and northern Japan), supporting the separation of *L. japonica* into three different species with additional evidence will be possible. The findings of the present study can be a stepping stone to developing a powerful indicator, such as a Rosetta Stone, for monitoring the northward migration of marine invertebrates as a result of global warming in the northwestern Pacific Ocean.

## Materials and methods

### Sample collection

A total of 342 *L*. *japonica* samples were collected from 12 locations in the intertidal coasts on the Korean Peninsula and Japanese Archipelago in the northwestern Pacific Ocean (Fig. 1; Table S1). After collection, the entire chiton body was immediately fixed in 100% ethanol and stored at -20 °C until total cellular DNA extraction.

### PCR amplification and sequencing

Genomic DNA was extracted from the foot muscle tissue using an Exgene Tissue SV kit (GeneAll, South Korea) following the manufacturer’s protocol. A 635-bp fragment of mitochondrial *COI* was amplified using the previously reported primer sets LCO1490 (5′-GGT CAA CAA ATC ATA AAG ATA TTG G-3′) and HCO2198 (5′-TAA ACT TCA GGG TGA CCA AAA AAT CA-3′)^32^. These primers, which were developed for use in invertebrates, can be used for PCR amplification of a partial mitochondrial *COI* gene fragment. A 506-bp sequence of the mitochondrial *16S rRNA* was also amplified using the previously reported primer sets 16Sa (5′-CGC CTG TTT ATC AAA AAC AT-3′) and 16Sb (5′-CTC CGG TTT GAA CTC AGA TCA-3′)^33^. PCR amplifications were carried out in a 50-μl volume reaction containing 3 μl genomic DNA, 5 μl 10 × PCR buffer, 4 μl dNTPs mixture (2.5 mM), 1 μl of each primer (20 μM), 1 μl *Taq* DNA polymerase (5 U/μl) and 35 μl double-distilled water. Thermocycling consisted of an initial denaturing step of 2 min at 94 °C, 30 cycles of denaturation for 30 s at 94 °C, annealing for 30 s at 55 °C to 60 °C, and extension for 30 s at 72 °C, and a final extension step of 5 min at 72 °C. The amplified products were purified using HiYield™ GEL/PCR DNA Extraction Kit (RBC Co., South Korea), and then sequenced by the commercial sequencing service company Genotech Co. (Daejeon, South Korea) using an ABI PRISM BigDye terminator system and an ABI 3700 analyzer. The novel sequences obtained in this study were deposited in the NCBI GenBank under accession numbers KT932836 − KT932912 for *COI* and KT932913 − KT932935 for *16S rRNA*.

### Genetic diversity and population genetic analyses

After trimming the obtained sequences using BioEdit^34^, to perform genetic diversity and population genetic analyses, 106 *COI* and 34 *16S rRNA* haplotype sequences were aligned with Clustal X^35^ (Data S1, S2). The numbers of polymorphic sites and haplotypes, haplotype diversity, and nucleotide diversity (Tables S1, S4, S5, S8) were estimated for each population using the program DnaSP v.6.0^36^. AMOVA was conducted using ARLEQUIN v.3.5^37^ to partition the genetic variance within and among populations. Furthermore, a statistical parsimony haplotype network was constructed with a 95% connection limit using TCS v.1.2.1^38^, and was used to assess the genealogical relationship by constructing the *COI* and *16S rRNA* haplotype networks. To evaluate and visualize the geographic genetic structure among populations, PCoA based on the *COI* and *16S rRNA* haplotypes were conducted using DARwin v.6.0.9^39^, which ordinated genetic distance estimates calculated using the haplotype data from this study.

### Phylogenetic analyses

Phylogenetic analyses using the *COI* and *16S rRNA* sequences based on the sequence alignment sets of 106 *COI* haplotypes (Data S1) and 34 *16S rRNA* haplotypes (Data S2) obtained in this study were performed using three different tree reconstruction algorithms: ML, BI, and NJ methods. In the ML tree, model selection in the IQ-Tree software package (http://www.iqtree.org) tested and selected the substitution model HKY + F + I as the best-fit model under Bayesian information criterion. The tree was computed from 1000 ultrafast bootstrap replicates using the IQ-Tree website (http://iqtree.cibiv.univie.ac.at)^40^. For BI, the models of sequence evolution for each gene were selected with jModeltest Ver. 2^41^, and were then trained in MrBayes (HKY + F + I). Each dataset was run for 10,000,000 iterations with a sample frequency of 1,000 iterations. After determining that the Markov Chain Monte Carlo (MCMC) runs reached a stationary level, the first 25% of the iterations (“burn-in” fraction) were discarded. The NJ tree was reconstructed using MEGA X^42^, and the confidence value for each node was calculated from 1000 bootstrap replicates. The haplotype sequences used for the phylogenetic analyses are listed in Tables S2 and S6, which include the sequences collected from the Zhejiang Province (ZJ) samples in southern China^8^ and Miyagi (MY) and Ehime (EH) samples from Japan retrieved from the NCBI GenBank. The outgroup species employed for the phylogenetic analyses were *A. spinosa* for the *COI* dataset and *A. echinata* for the *16S rRNA* dataset. Additionally, a phylogenetic network for each sequence alignment dataset was generated via the neighbor net algorithm^43^.

### DNA barcoding gap analyses

The analyses of barcoding gaps based on *COI* and *16S rRNA* were conducted using the online version of ABGD^44^ (https://bioinfo.mnhn.fr/abi/public/abgd/abgdweb.html) to generate distance histograms, distance ranks, and automatic partitions. These analyses were conducted using the Kimura 2-P distance matrix^45^, and two different parameters: the range of prior intraspecific divergence from *P*_min_ (0.001) to *P*_max_ (0.1), and relative gap width (*X* = 1.5). Furthermore, we implemented two DNA taxonomy approaches to evaluate for the presence of cryptic species on the basis of *COI*: the GMYC approach^46^ and a bPTP^47^. The GMYC approach was applied to an ultrametric tree produced by BEAST 2.6.0^48^ with the Splits package (http://splits.r-forge.r-project.org). It is a process-based approach to detect the threshold at which processes within a species (i.e., coalescence) shift to the processes between species (i.e., speciation and extinction) in a gene tree. The bPTP was also performed to infer putative species boundaries on a given phylogenetic input tree. Unlike the GMYC model, the bPTP model requires a bifurcated phylogenetic tree rather than an ultrametric tree, and it can model speciation or branching events in terms of the number of substitutions. We used the following parameters for bPTP: MCMC 500,000 generations, 100 thinning, 10% initial iterations burn-in, and assessed convergence in each case to ensure the reliability of the results.

### Description of *Liolophura koreana*, sp. nov. and *L. sinensis*, sp. nov.

All samples examined in this study were deposited in the sample collection of Institute for Phylogenomics and Evolution, Kyungpook National University, Daegu, South Korea. The 25 type specimens for *L. koreana* sp. nov. are deposited under voucher nos. LEGOM040501–LEGOM040525. In the case of *L. sinensis* sp. nov., we could not designate the type specimens because only the sample photos and *COI* barcoding marker were examined without specimens. The *L. sinensis* samples that were analyzed are kept in College of Life and Environmental Sciences, Wenzhou University, Zhejiang Province, China. We used the morphological terminology of Schwabe^49^. In the description of the new species, the parentheses indicate character states for intraspecific variations. The chitons were observed with a Leica M205 C stereo microscope (Leica Camera AG, Germany). The images of the chitons were captured with a Leica MC190 HD camera mounted on a Leica M205 C stereo microscope (Leica Camera AG, Germany) and were produced with Leica Application Suite version 4.12.0 (Leica Camera AG, Germany). Detailed morphological structures of the dorsal side of the valves and perinotum were imaged using Field Emission Scanning Electron Microscopy (FE-SEM) (SU8220, Hitachi, Tokyo, Japan). Final panels were prepared in Adobe Photoshop CS6 (Adobe Systems Incorporated, San Jose, USA).

### Demographic inferences and divergence time estimation

Historical demographic changes were estimated based on two different approaches. First, Tajima’s *D*^50^ and Fu’s *Fs*^51^ statistical methods were used via ARLEQUIN v.3.5^36^ to test for the historical signature of population expansion under mutation-drift equilibrium. Second, to examine whether *Liolophura* species underwent recent expansions or frequent migrations among neighbor demes, an MDA with the *COI* sequence data was performed to graphically indicate the observed distribution of pairwise differences between haplotypes using a model of demographic expansions^52^ using DnaSP v.6.0^36^. A BSP was computed to examine the historical demographic fluctuation since the time of the most recent common ancestor using BEAST 2.6.0^47^. The HKY model was selected as the best-fit nucleotide substitution model by jModelTest^41^ and mutation rates of 2.0 × 10^−8^^53,54^ under a strict molecular clock^55^ was used and MCMC was run for 30 million steps. Then, 10% of the iterations that had not reached convergence were burned, and a tree was sampled every 1,000 iterations. TRACER 1.7 program^56^ was used to construct the BSP^57^.

Divergence time estimation for the three phylogenetic lineages of *L*. *koreana*, sp. nov. (Lineage N), *L*. *japonica* (Lineage S1), and *L*. *sinensis*, sp. nov. (Lineage S2)*,* as done in Choi et al.^58^, was conducted based on *COI* haplotype sequences using BEAST 2.6.0.^48^. The divergence time was estimated using the strict molecular clock^55^ algorithm under the calibrated-Yule tree, which was calibrated using the earliest fossil record data of *Mopalia* (ca. 17.2 − 15 Ma)^59^ for the basal node (17 Ma; normal distribution); a “monophyly” option was chosen in the BEAUti 2 program (*Mopalia*, *COI* haplotypes of the *Liolophura* species). Posterior distributions of the parameter were estimated using 1,000,000 MCMC iterations and sampled every 1,000 iteration, after discarding the initial 20% of iterations as burn-in. We used HKY as the best-fit substitution model selected by jModelTest ver. 2^41^. Additionally, an effective population size was determined using Tracer 1.7^56^. TreeAnnotator 2.6.0.^60^ was used to produce a tree with maximum clade credibility and a median height after removing the initial 25% of iterations as burn-in. FigTree 1.4.2.^61^ was used to visualize the topology of the resultant consensus tree.

## Supplementary Information

Supplementary Information.

## Data Availability

The sequence data have been deposited to the NCBI GenBank database under the accession numbers KT932836 − KT932912 for *COI* and KT932913 − KT932935 for *16S rRNA*. The pre-processed datasets are available at Dryad Digital Repository, https://doi.org/10.5061/dryad.rr4xgxd7h, which are named as follows: Data S1, Nucleotide sequence alignment of 106 *COI* haplotypes of *L. japonica* with the outgroup *A. spinose*; Data S2, Nucleotide sequence alignment of 34 *16S rRNA* haplotypes of *L. japonica* with the outgroup *A. echinata*; Data S3, Nucleotide sequence alignment of 106 *COI* haplotypes of *L. japonica, L. koreana* sp. nov., and *L. sinensis* sp. nov., one *COI* haplotype of *L. tenuispinosa*, and 14 *COI* haplotypes of eight *Acanthopluera* congeneric species with an outgroup *Tonicia forbesii*.
