# Supplementary material for: Liolophura species discrimination with geographical distribution patterns and their divergence and expansion history on the northwestern Pacific coast
Source: Sci Rep. 2021 Sep 2;11:17602. doi: 10.1038/s41598-021-96823-5 (PMC8413323; doi:10.1038/s41598-021-96823-5)
Supplement: Supplementary file 1 — Supplementary Information. [file 41598_2021_96823_MOESM1_ESM.docx]

**Supplementary Information**

***Liolophura* species discrimination with geographical distribution patterns and their divergence and expansion history on the northwestern Pacific coast**

Eun Hwa Choi^1,2#^, Mi Yeong Yeo^1#^, Gyeongmin Kim^1,3#^, Bia Park^1,2#^, Cho Rong Shin^1^, Su Youn Baek^1,2^, and Ui Wook Hwang^1,2,4*^


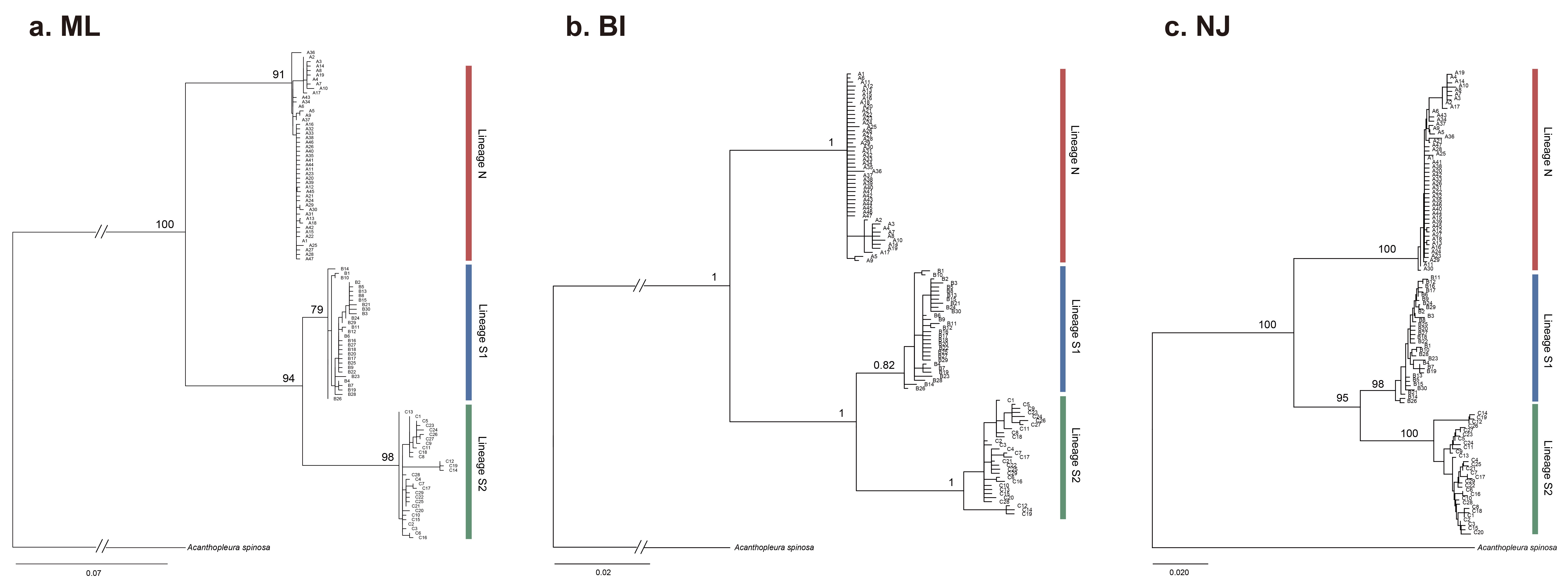


**Figure S1.** Phylogenetic trees reconstructed based on 106 *COI* haplotypes of *Liolophura japonica* using maximum likelihood (a; ML), Bayesian inference (b; BI), and neighbor joining (c; NJ) methods: BP in ML, BPP in BI, and BP in NJ. The resultant trees exhibit an identical topology, of which the node confidence values are depicted on the tree branches. *Acanthopleura spinosa* was used as an outgroup.


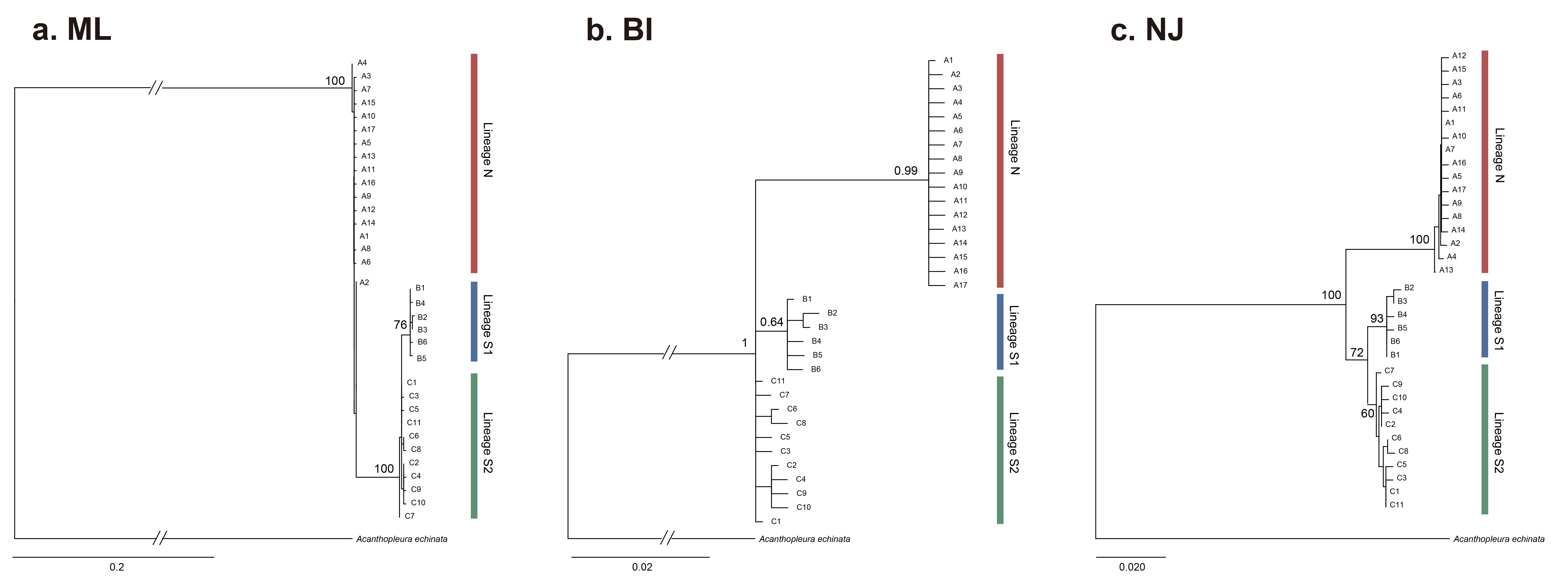


**Figure S2.** Phylogenetic tree reconstructed based on 34 *16S rRNA* haplotypes of *Liolophura japonica* using maximum likelihood (a; ML), Bayesian inference (b; BI), and neighbor joining (c; NJ) methods: BP in ML, BPP in BI, and BP in NJ. The resultant trees exhibit an identical topology, of which the node confidence values are depicted on the tree branches. *Acanthopleura echinata* was used as an outgroup.


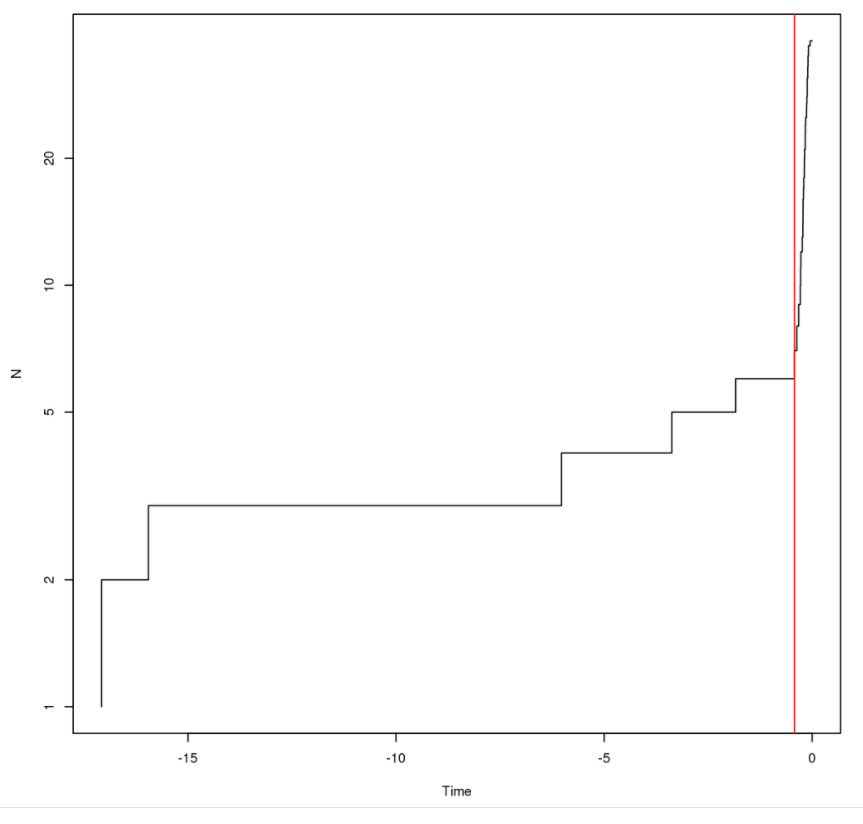

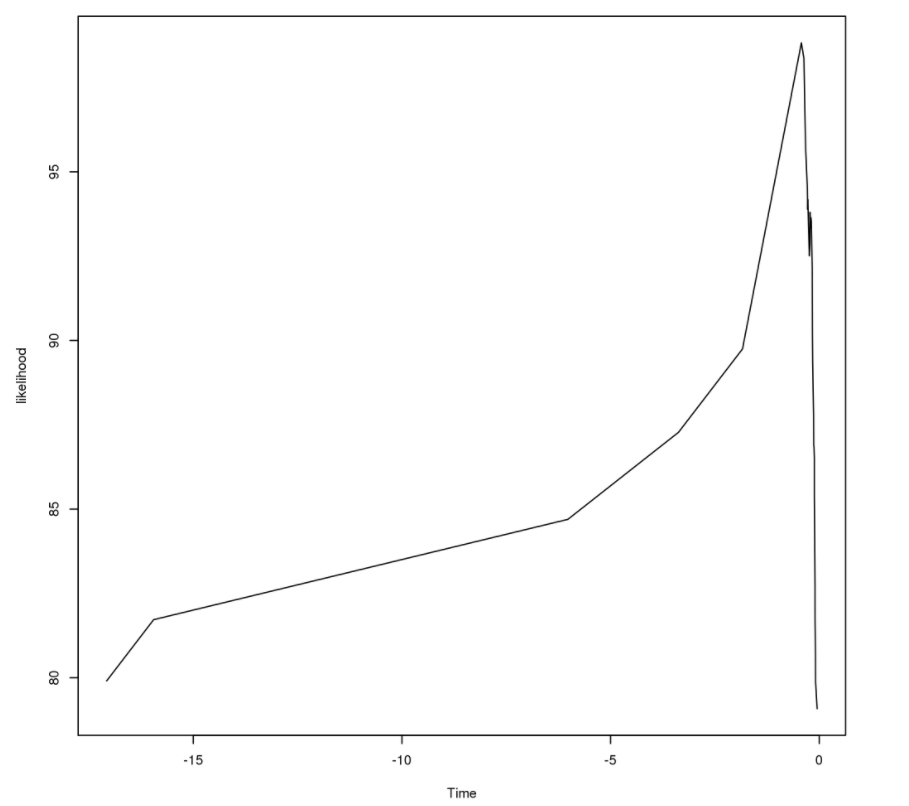

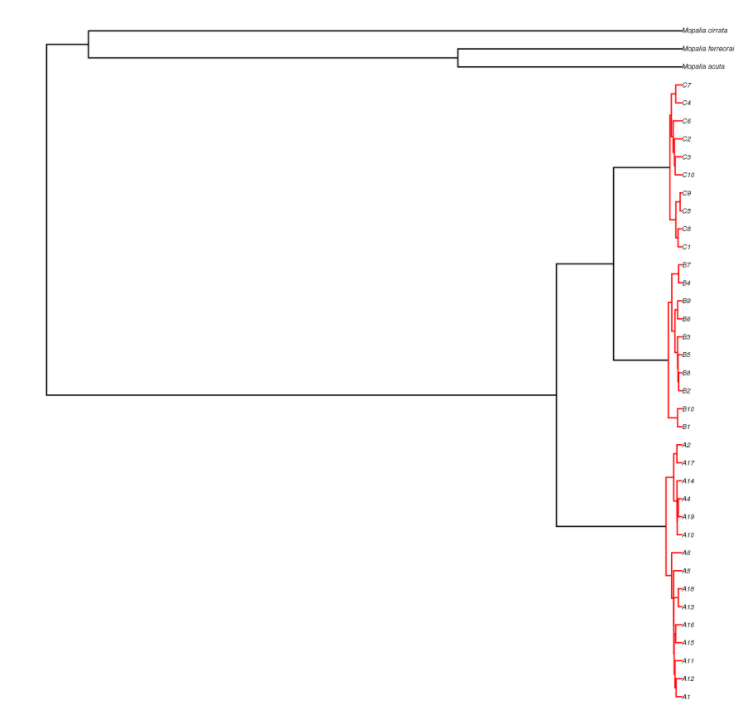


**Figure S3.** The general mixed Yule coalescent (GMYC) approach based on *COI* of *Liolophura japonica*. GMYC was applied to an ultrametric tree produced by BEAST 2.6.0^47^ with the Splits package ([http://splits.r-forge.r-project.org](http://splits.r-forge.r-project.org/)).


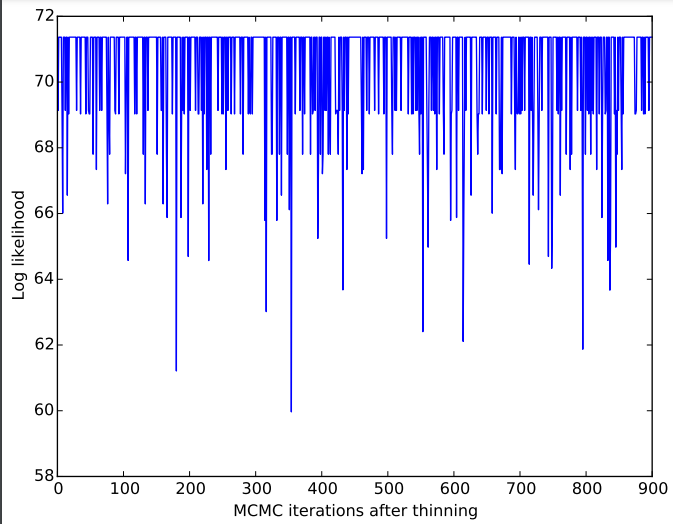

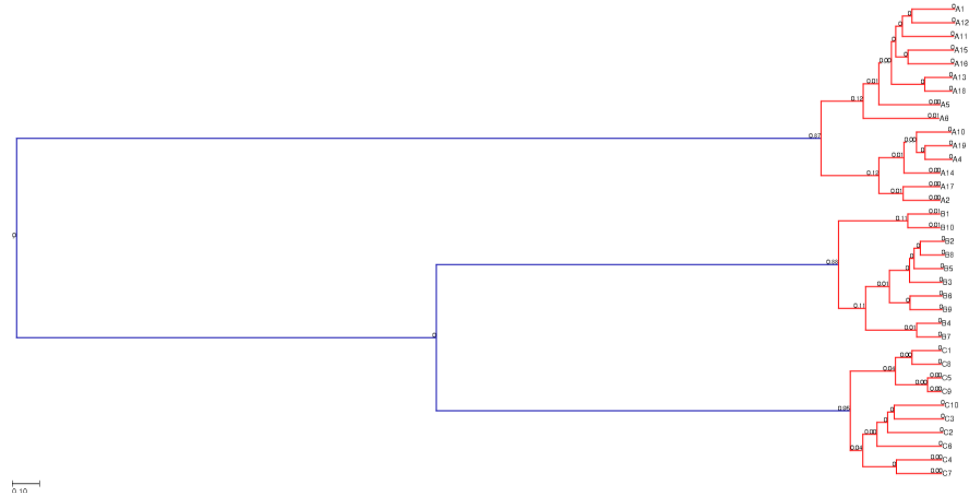


**Figure S4.** Bayesian implementation of Poisson Tree Processes model (bPTP). The bPTP was performed to infer putative species boundaries on a given phylogenetic input tree. We used the following parameters for bPTP: MCMC 500,000 generations, 100 thinning, 10% initial iterations burn-in, and assessed convergence in each case to ensure the reliability of the results.


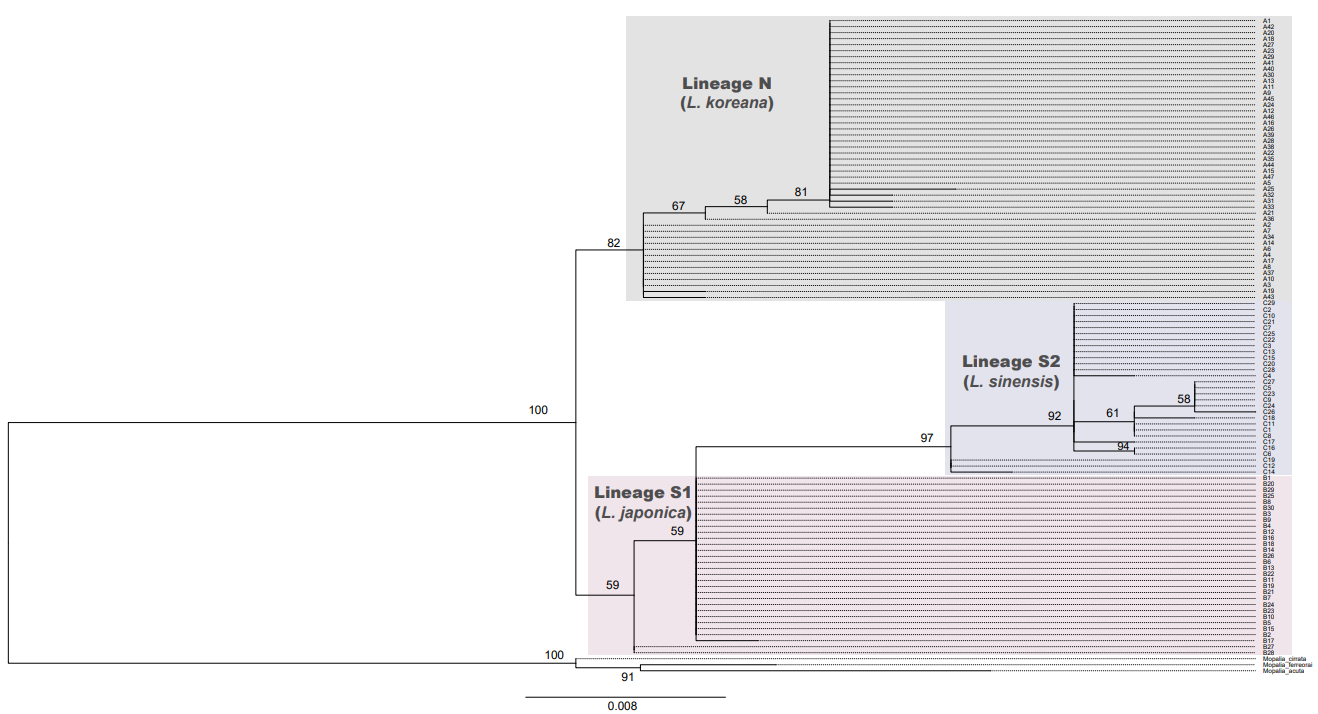


**Figure S5.** A maximum likelihood tree reconstructed with 22 polymorphic sites which were found only in the first and second codon positions of COI using IQ-TREE2. The best-fit evolutionary model TN+F+G4 was selected and adapted. In total, 1,000 bootstrap replicates were used for node confidence values. The three *Mopalia* species were used as outgroups.


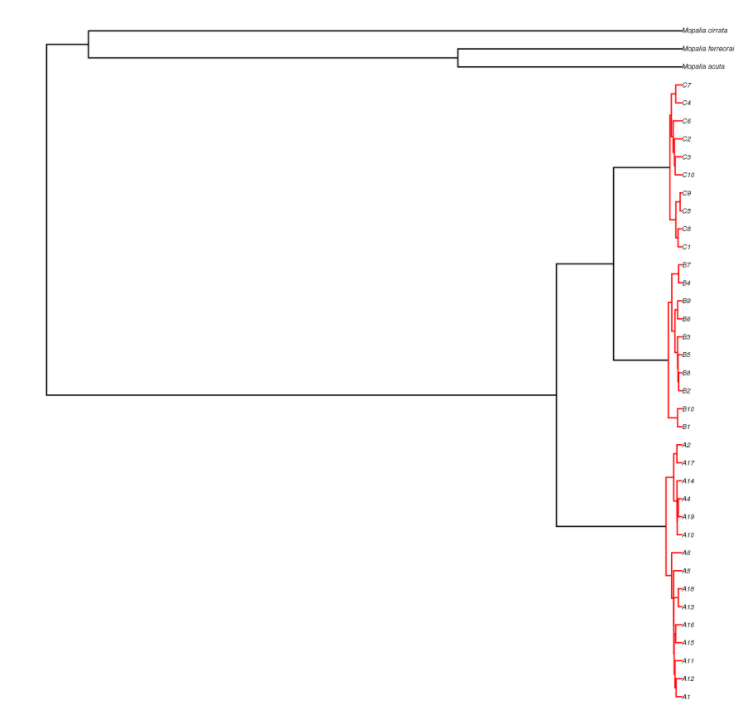

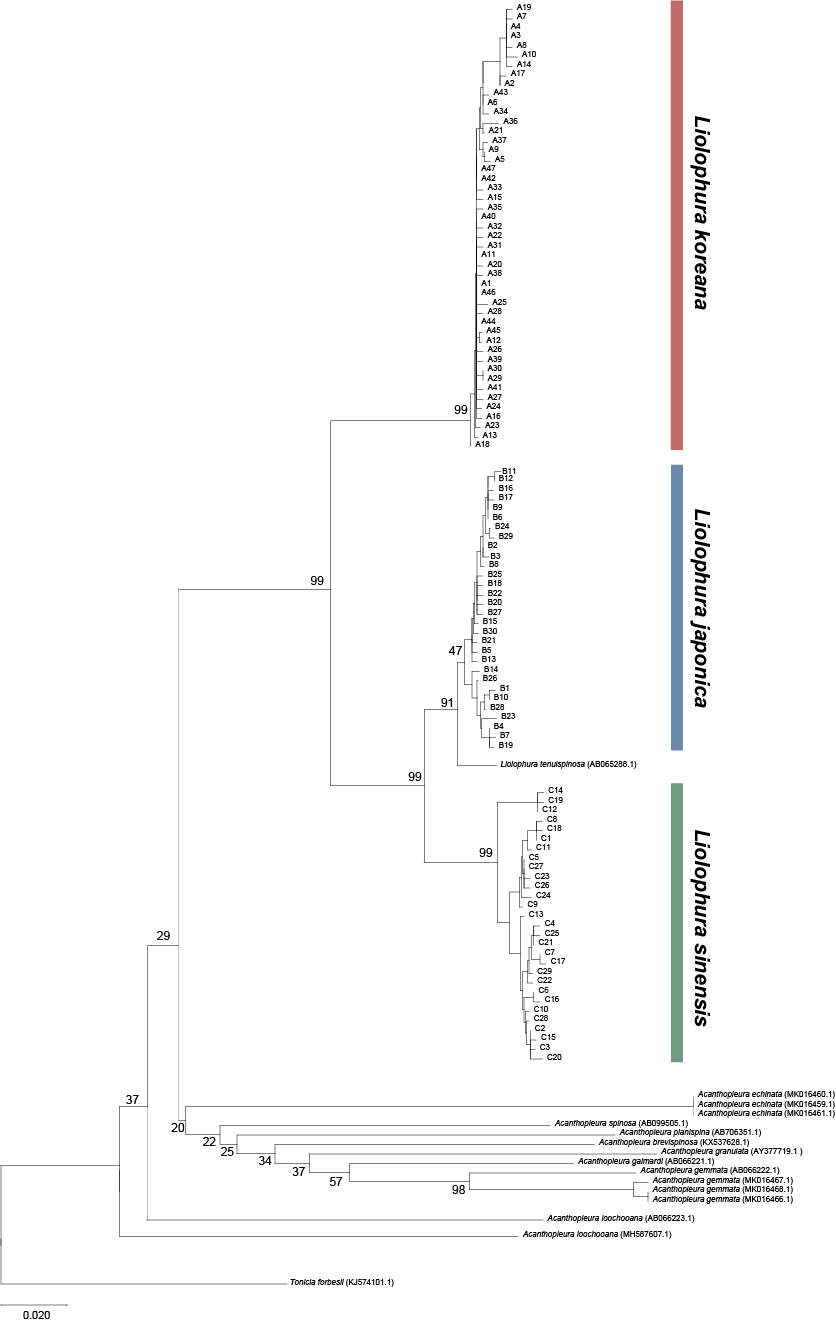
**Figure S6.** Neighbor joining tree reconstructed based on 106 *COI* haplotypes of *Liolophura japonica, Liolophura koreana* sp. nov., and *Liolophura sinensis* sp. nov., one *COI* haplotype of *Liolophura tenuispinosa*, and 14 *COI* haplotypes of eight *Acanthopluera* congeneric species as an outgroup *Tonicia forbesii*.


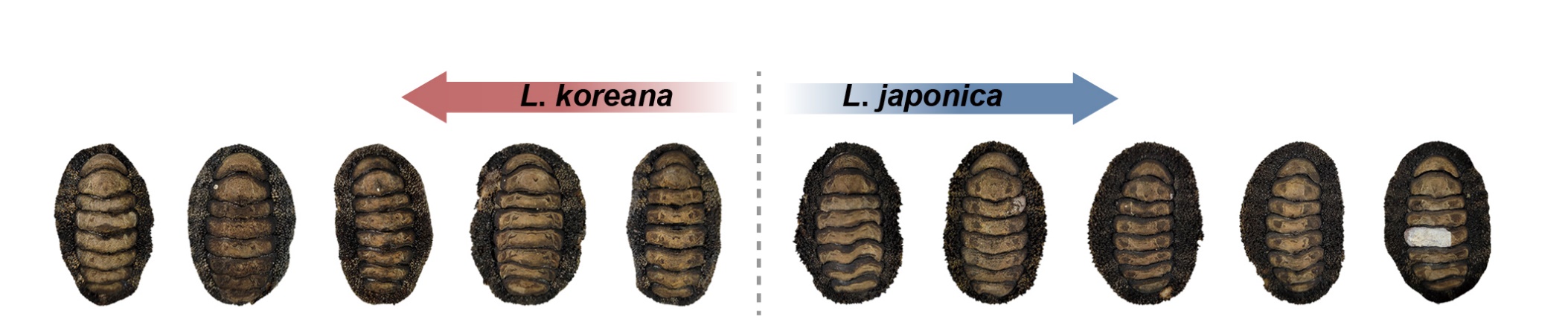


**Figure S7.** A series of photos showing morphological variations of *Liolophura* *koreana*, sp. nov. and *Liolophura* *japonica*.


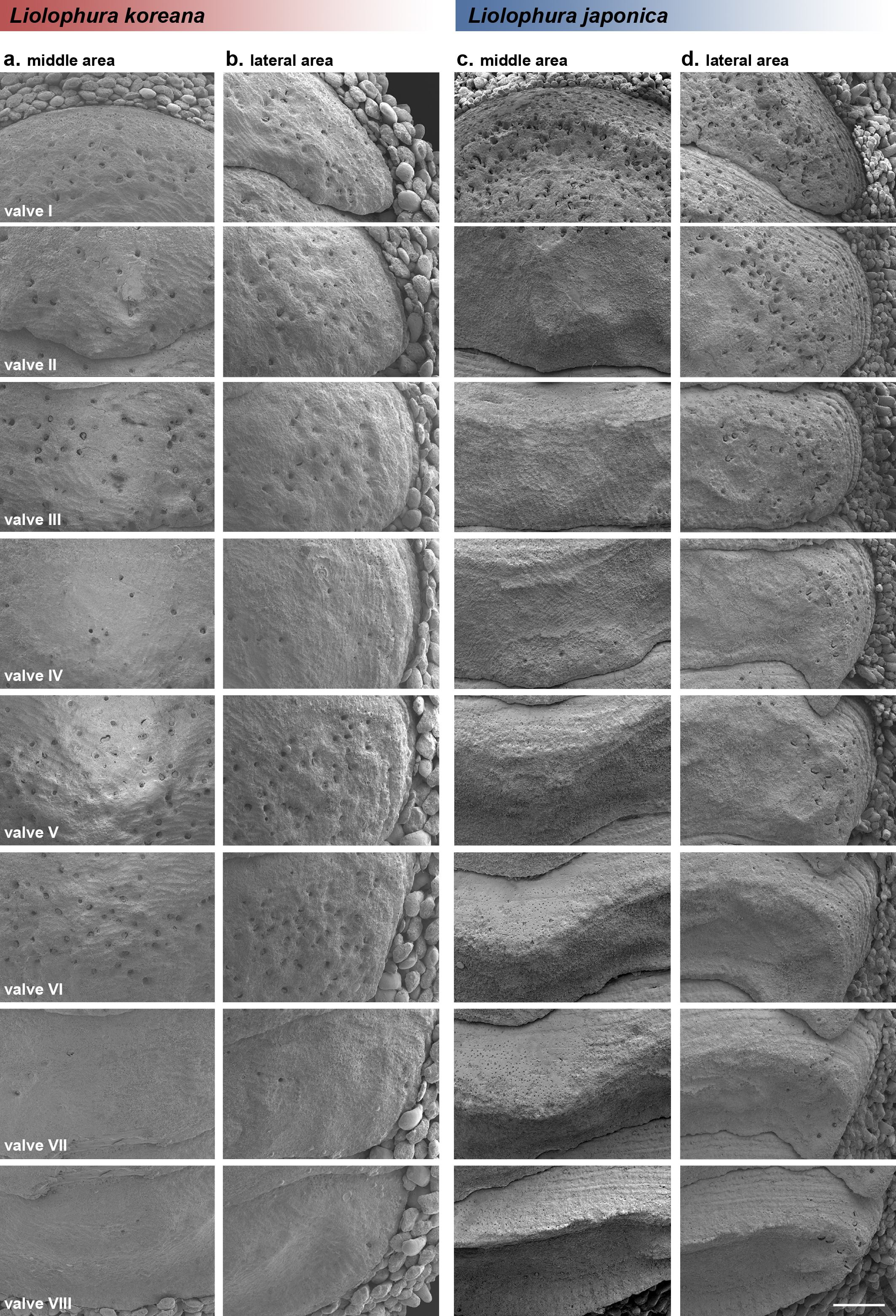


**Figure S8.** Microstructural comparison of the tegmentum in the middle and lateral areas between *Liolophura koreana*, sp. nov. and *Liolophura japonica* using the FE-SEM. **(a, b)** Middle and lateral areas on the tegmentum of the holotype of *L*. *koreana*. **(c, d)** Middle and lateral areas on the tegmentum of *L*. *japonica*. The scale bar marks 1.0 mm.


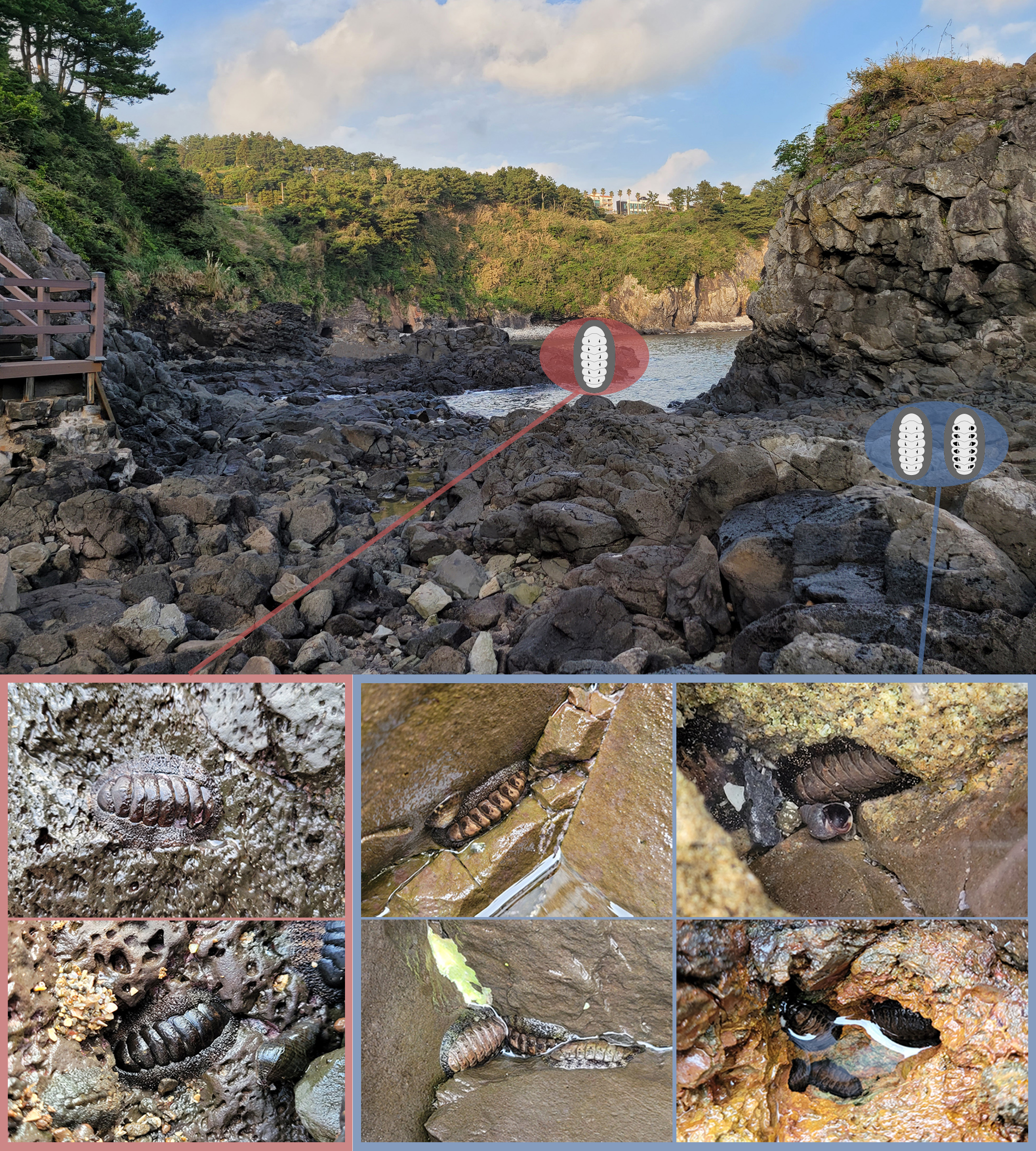


**Figure S9.** The landscape photos showing microhabitat differences between *Liolophura koreana*, sp. nov. and *Liolophura* *japonica*, which were taken from Seogwipo-si, Jeju Island, South Korea by Bia Park and Cho Rong Shin. *L*. *japonica* are mainly found in quiet spots on inner shore in coastal areas of the northwestern Pacific, while *L*. *koreana* appear in a wide range of environments, even around directly exposed areas where sea waves strike.


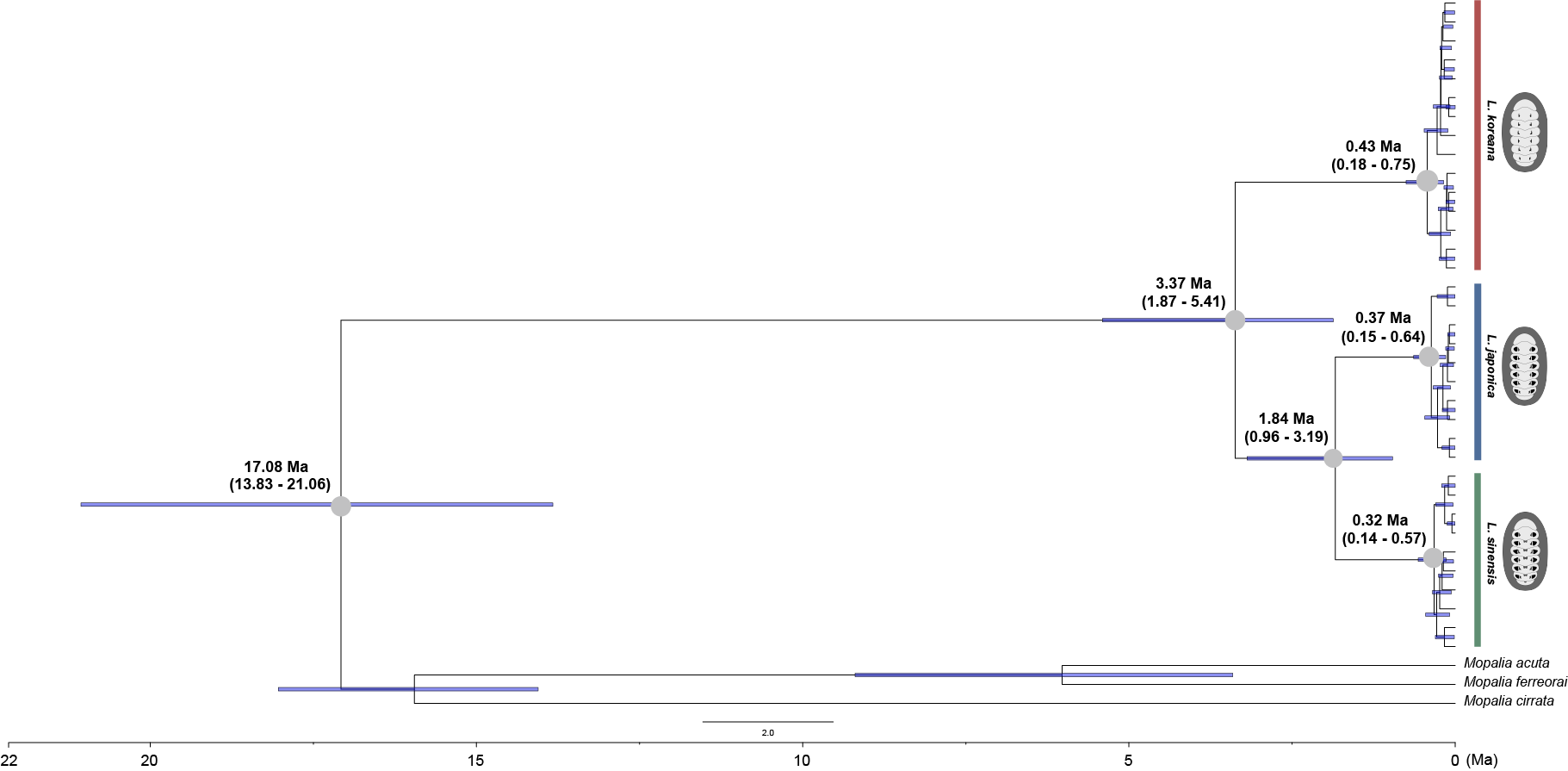


**Figure S10.** Time-calibrated Bayesian tree reconstructed with 106 *COI* haplotypes of *Liolophura japonica* using the BEAST 2.6.0. program. The haplotype information used here is listed in Supplementary Table 2.

**Table S1.** Summary of sampling locations, genetic diversity indices and references for *COI* haplotypes of *Liolophura japonica* inhabiting the northwestern Pacific.

| **Country** | **Sampling location** | **Latitude** | ***N*** | ***N*_H_** | ***h*** | ***π*** | **References** |
| --- | --- | --- | --- | --- | --- | --- | --- |
| **South Korea** | **Ulleungdo (UL)** | 37.46°N | 104 | 18 | 0.527 | 0.00258 | Present study |
|  | **Dokdo (DD)** | 37.24°N | 23 | 8 | 0.632 | 0.00410 | Present study |
|  | **Pohang (PH)** | 36.02°N | 35 | 8 | 0.526 | 0.00171 | Present study |
|  | **Busan (BS)** | 35.10°N | 16 | 2 | 0.125 | 0.00079 | Present study |
|  | **Tongyeong (TY)** | 34.81°N | 31 | 14 | 0.735 | 0.02506 | Present study |
|  | **Yeosu (YS)** | 34.72°N | 14 | 7 | 0.692 | 0.00158 | Present study |
|  | **Geojedo (GJ)** | 34.70°N | 28 | 13 | 0.683 | 0.02307 | Present study |
|  | **Wando (WD)** | 34.30°N | 12 | 3 | 0.621 | 0.04581 | Present study |
|  | **Sinan (SD)** | 34.34°N | 10 | 3 | 0.378 | 0.00158 | Present study |
|  | **Jeju (JJ)** | 33.24°N | 14 | 3 | 0.582 | 0.04456 | Present study |
| **Japan** | **Miyagi(MY)** | 38.32°N | 1 | 1 | n. s. | n. s. | Retrieved |
|  | **Tottori(TT)** | 35.53°N | 30 | 15 | 0.814 | 0.04301 | Present study |
|  | **Tsushima(TS)** | 34.45°N | 25 | 20 | 0.963 | 0.01090 | Present study |
|  | **Ehime(EH)** | 33.70°N | 1 | 1 | n. s. | n. s. | Retrieved |
| **China** | **Zhejiang (ZJ)** | 27.02–28.00°N | 125 | 28 | 1.000 | 0.00975 | Wu et al. 2018 |
| **Total** | | | **469** | **106** | **0.808** | **0.04936** |  |
| ***N***: number of sampled specimens; ***N*_H_**: number of observed haplotypes; ***h***: haplotype diversity; ***π***: nucleotide diversity; n. s.: not significant | | | | | | | |

**Table S2.** Summary information of 106 *COI* haplotypes observed from 469 individuals of *Liolophura japonica* inhabiting the northwestern Pacific.

| **No.** | **Species** | **Country** | **Location** | **Accession No.** | **Haplotype** | **Reference** |
| --- | --- | --- | --- | --- | --- | --- |
| **Ingroup** | | | | | | |
| 01 | *Liolophura japonica* | South Korea  Japan | All 10 locations  Totori | KT932836 | A1 | Present study |
| 02 | *Liolophura japonica* | South Korea | Dokdo/Ullenugd/Pohang/  Busan/Sinan/Tongyeong/Geojedo | KT932837 | A2 | Present study |
| 03 | *Liolophura japonica* | South Korea | Dokdo | KT932838 | A3 | Present study |
| 04 | *Liolophura japonica* | South Korea | Dokdo/Ulleungdo | KT932839 | A4 | Present study |
| 05 | *Liolophura japonica* | South Korea | Dokdo/Ulleungdo | KT932840 | A5 | Present study |
| 06 | *Liolophura japonica* | South Korea | Dokdo/Ulleungdo  Pohang/Tongyeong | KT932841 | A6 | Present study |
| 07 | *Liolophura japonica* | South Korea | Dokdo | KT932842 | A7 | Present study |
| 08 | *Liolophura japonica* | South Korea | Dokdo | KT932843 | A8 | Present study |
| 09 | *Liolophura japonica* | South Korea | Ulleungdo/Pohang/Yeosu | KT932844 | A9 | Present study |
| 10 | *Liolophura japonica* | South Korea | Ulleungdo | KT932845 | A10 | Present study |
| 11 | *Liolophura japonica* | South Korea | Ulleungdo/Pohang, Tongyeong/Geojedo/Yeosu | KT932846 | A11 | Present study |
| 12 | *Liolophura japonica* | South Korea | Ulleungdo | KT932847 | A12 | Present study |
| 13 | *Liolophura japonica* | South Korea | Ulleungdo | KT932848 | A13 | Present study |
| 14 | *Liolophura japonica* | South Korea | Ulleungdo | KT932849 | A14 | Present study |
| 15 | *Liolophura japonica* | South Korea | Ulleungdo | KT932850 | A15 | Present study |
| 16 | *Liolophura japonica* | South Korea | Ulleungdo | KT932851 | A16 | Present study |
| 17 | *Liolophura japonica* | South Korea | Ulleungdo | KT932852 | A17 | Present study |
| 18 | *Liolophura japonica* | South Korea | Ulleungdo | KT932853 | A18 | Present study |
| 19 | *Liolophura japonica* | South Korea | Ulleungdo | KT932854 | A19 | Present study |
| 20 | *Liolophura japonica* | South Korea | Ulleungdo | KT932855 | A20 | Present study |
| 21 | *Liolophura japonica* | South Korea | Ulleungdo | KT932856 | A21 | Present study |
| 22 | *Liolophura japonica* | South Korea | Pohang | KT932857 | A22 | Present study |
| 23 | *Liolophura japonica* | South Korea | Pohang | KT932858 | A23 | Present study |
| 24 | *Liolophura japonica* | South Korea | Pohang | KT932859 | A24 | Present study |
| 25 | *Liolophura japonica* | South Korea | Yeosu | KT932860 | A25 | Present study |
| 26 | *Liolophura japonica* | South Korea | Yeosu | KT932861 | A26 | Present study |
| 27 | *Liolophura japonica* | South Korea | Yeosu/Geojedo | KT932862 | A27 | Present study |
| 28 | *Liolophura japonica* | South Korea | Yeosu | KT932863 | A28 | Present study |
| 29 | *Liolophura japonica* | South Korea | Tongyeong | KT932864 | A29 | Present study |
| 30 | *Liolophura japonica* | South Korea | Tongyeong | KT932865 | A30 | Present study |
| 31 | *Liolophura japonica* | South Korea | Tongyeong | KT932866 | A31 | Present study |
| 32 | *Liolophura japonica* | South Korea | Tongyeong | KT932867 | A32 | Present study |
| 33 | *Liolophura japonica* | South Korea | Tongyeong | KT932868 | A33 | Present study |
| 34 | *Liolophura japonica* | South Korea | Tongyeong | KT932869 | A34 | Present study |
| **No.** | **Species** | **Country** | **Location** | **Accession No.** | **Haplotype** | **Reference** |
| 35 | *Liolophura japonica* | South Korea | Tongyeong | KT932870 | A35 | Present study |
| 36 | *Liolophura japonica* | South Korea | Geojedo | KT932871 | A36 | Present study |
| 37 | *Liolophura japonica* | South Korea | Geojedo | KT932872 | A37 | Present study |
| 38 | *Liolophura japonica* | South Korea | Geojedo | KT932873 | A38 | Present study |
| 39 | *Liolophura japonica* | South Korea | Geojedo | KT932874 | A39 | Present study |
| 40 | *Liolophura japonica* | South Korea | Geojedo | KT932875 | A40 | Present study |
| 41 | *Liolophura japonica* | Japan | Tsushima | KT932876 | A41 | Present study |
| 42 | *Liolophura japonica* | Japan | Totori | KT932877 | A42 | Present study |
| 43 | *Liolophura japonica* | Japan | Totori | KT932878 | A43 | Present study |
| 44 | *Liolophura japonica* | South Korea  Japan | Sinan  Totori | KT932879 | A44 | Present study |
| 45 | *Liolophura japonica* | Japan | Totori | KT932880 | A45 | Present study |
| 46 | *Liolophura japonica* | Japan | Totori | KT932881 | A46 | Present study |
| 47 | *Liolophura japonica* | Japan | Miyagi | AB064987 | A47 | unpublished |
| 48 | *Liolophura japonica* | South Korea Japan | Tongyeong/Wando  Totori | KT932883 | B1 | Present study |
| 49 | *Liolophura japonica* | South Korea Japan | Tongyeong/Jeju  Tsushima/Totori, | KT932884 | B2 | Present study |
| 50 | *Liolophura japonica* | South Korea | Tongyeong | KT932885 | B3 | Present study |
| 51 | *Liolophura japonica* | South Korea Japan | Geojedo/Wando/Jeju  Totori | KT932886 | B4 | Present study |
| 52 | *Liolophura japonica* | South Korea | Geojedo | KT932887 | B5 | Present study |
| 53 | *Liolophura japonica* | South Korea  Japan | Geojedo  Tsushima/Totori | KT932888 | B6 | Present study |
| 54 | *Liolophura japonica* | South Korea | Geojedo | KT932889 | B7 | Present study |
| 55 | *Liolophura japonica* | Japan | Tsushima | KT932890 | B8 | Present study |
| 56 | *Liolophura japonica* | Japan | Tsushima | KT932891 | B9 | Present study |
| 57 | *Liolophura japonica* | Japan | Tsushima | KT932892 | B10 | Present study |
| 58 | *Liolophura japonica* | Japan | Tsushima | KT932893 | B11 | Present study |
| 59 | *Liolophura japonica* | Japan | Tsushima | KT932894 | B12 | Present study |
| 60 | *Liolophura japonica* | Japan | Tsushima | KT932895 | B13 | Present study |
| 61 | *Liolophura japonica* | Japan | Tsushima | KT932896 | B14 | Present study |
| 62 | *Liolophura japonica* | Japan | Tsushima | KT932897 | B15 | Present study |
| 63 | *Liolophura japonica* | Japan | Tsushima | KT932898 | B16 | Present study |
| 64 | *Liolophura japonica* | Japan | Tsushima | KT932899 | B17 | Present study |
| 65 | *Liolophura japonica* | Japan | Tsushima | KT932900 | B18 | Present study |
| 66 | *Liolophura japonica* | Japan | Tsushima | KT932901 | B19 | Present study |
| 67 | *Liolophura japonica* | Japan | Tsushima | KT932902 | B20 | Present study |
| 68 | *Liolophura japonica* | Japan | Tsushima | KT932903 | B21 | Present study |
| 69 | *Liolophura japonica* | Japan | Tsushima | KT932904 | B22 | Present study |
| 70 | *Liolophura japonica* | Japan | Tsushima | KT932905 | B23 | Present study |
| 71 | *Liolophura japonica* | Japan | Tsushima | KT932906 | B24 | Present study |
| **No.** | **Species** | **Country** | **Location** | **Accession No.** | **Haplotype** | **Reference** |
| 72 | *Liolophura japonica* | Japan | Tottori | KT932907 | B25 | Present study |
| 73 | *Liolophura japonica* | Japan | Tottori | KT932908 | B26 | Present study |
| 74 | *Liolophura japonica* | Japan | Tottori | KT932909 | B27 | Present study |
| 75 | *Liolophura japonica* | Japan | Tottori | KT932910 | B28 | Present study |
| 76 | *Liolophura japonica* | Japan | Tottori | KT932911 | B29 | Present study |
| 77 | *Liolophura japonica* | Japan | Ehime | AB064986 | B30 | Unpublished |
| 78 | *Liolophura japonica* | China | Zhejiang | MF716580 | C1 | Wu et al., 2018 |
| 79 | *Liolophura japonica* | China | Zhejiang | MF774377 | C2 | Wu et al., 2018 |
| 80 | *Liolophura japonica* | China | Zhejiang | MF774378 | C3 | Wu et al., 2018 |
| 81 | *Liolophura japonica* | China | Zhejiang | MF774379 | C4 | Wu et al., 2018 |
| 82 | *Liolophura japonica* | China | Zhejiang | MF774380 | C5 | Wu et al., 2018 |
| 83 | *Liolophura japonica* | China | Zhejiang | MF774382 | C6 | Wu et al., 2018 |
| 84 | *Liolophura japonica* | China | Zhejiang | MF774383  MG203946 | C7 | Wu et al., 2018  unpublished |
| 85 | *Liolophura japonica* | China | Zhejiang | MF774384 | C8 | Wu et al., 2018 |
| 86 | *Liolophura japonica* | China | Zhejiang | MF774385 | C9 | Wu et al., 2018 |
| 87 | *Liolophura japonica* | China | Zhejiang | MF774386 | C10 | Wu et al., 2018 |
| 88 | *Liolophura japonica* | China | Zhejiang | MF774387 | C11 | Wu et al., 2018 |
| 89 | *Liolophura japonica* | China | Zhejiang | MF774388 | C12 | Wu et al., 2018 |
| 90 | *Liolophura japonica* | China | Zhejiang | MF774389 | C13 | Wu et al., 2018 |
| 91 | *Liolophura japonica* | China | Zhejiang | MF774390 | C14 | Wu et al., 2018 |
| 92 | *Liolophura japonica* | China | Zhejiang | MF774391 | C15 | Wu et al., 2018 |
| 93 | *Liolophura japonica* | China | Zhejiang | MF774392 | C16 | Wu et al., 2018 |
| 94 | *Liolophura japonica* | China | Zhejiang | MF774393 | C17 | Wu et al., 2018 |
| 95 | *Liolophura japonica* | China | Zhejiang | MF774394 | C18 | Wu et al., 2018 |
| 96 | *Liolophura japonica* | China | Zhejiang | MF774395 | C19 | Wu et al., 2018 |
| 97 | *Liolophura japonica* | China | Zhejiang | MF774396 | C20 | Wu et al., 2018 |
| 98 | *Liolophura japonica* | China | Zhejiang | MF774397 MF774381 | C21 | Wu et al., 2018 |
| 99 | *Liolophura japonica* | China | Zhejiang | MF774398 | C22 | Wu et al., 2018 |
| 100 | *Liolophura japonica* | China | Zhejiang | MF774399 | C23 | Wu et al., 2018 |
| 101 | *Liolophura japonica* | China | Zhejiang | MF774400 | C24 | Wu et al., 2018 |
| 102 | *Liolophura japonica* | China | Zhejiang | MF774401 | C25 | Wu et al., 2018 |
| 103 | *Liolophura japonica* | China | Zhejiang | MF774402 | C26 | Wu et al., 2018 |
| 104 | *Liolophura japonica* | China | Zhejiang | MF774403 | C27 | Wu et al., 2018 |
| 105 | *Liolophura japonica* | China | Zhejiang | MF774404 | C28 | Wu et al., 2018 |
| 106 | *Liolophura japonica* | China | Zhejiang | MF774405 | C29 | Wu et al., 2018 |
| **Outgroup** | | | | | | |
| 107 | *Acanthopleura spinosa* | Japan | Unknown | AB099505 | - | Unpublished |

**Table S3.** Geographical distribution along populations of 106 *COI* haplotypes observed from 469 individuals of *Liolophura japonica* inhabiting the northwestern Pacific.

| **Haplotype** |  | **South Korea** | | | | | | | | | |  | **Japan** | | | |  | **China** |  | **Total** |
| --- | --- | --- | --- | --- | --- | --- | --- | --- | --- | --- | --- | --- | --- | --- | --- | --- | --- | --- | --- | --- |
|  |  | **DD** | **UL** | **PH** | **BS** | **TY** | **GJ** | **YS** | **WD** | **SA** | **JJ** |  | **TS** | **TT** | **MY** | **EH** |  | **ZJ** |  |  |
| A1 |  | 14 | 71 | 24 | 15 | 16 | 16 | 8 | 5 | 8 | 8 |  |  | 13 |  |  |  |  |  | 198 |
| A2 |  | 2 | 3 | 3 | 1 | 1 | 1 |  |  | 1 |  |  |  |  |  |  |  |  |  | 12 |
| A3 |  | 1 |  |  |  |  |  |  |  |  |  |  |  |  |  |  |  |  |  | 1 |
| A4 |  | 2 | 8 |  |  |  |  |  |  |  |  |  |  |  |  |  |  |  |  | 10 |
| A5 |  | 1 | 1 |  |  |  |  |  |  |  |  |  |  |  |  |  |  |  |  | 2 |
| A6 |  | 1 | 1 | 1 |  | 1 |  |  |  |  |  |  |  |  |  |  |  |  |  | 4 |
| A7 |  | 1 |  |  |  |  |  |  |  |  |  |  |  |  |  |  |  |  |  | 1 |
| A8 |  | 1 |  |  |  |  |  |  |  |  |  |  |  |  |  |  |  |  |  | 1 |
| A9 |  |  | 6 | 1 |  |  |  | 1 |  |  |  |  |  |  |  |  |  |  |  | 8 |
| A10 |  |  | 1 |  |  |  |  |  |  |  |  |  |  |  |  |  |  |  |  | 1 |
| A11 |  |  | 1 | 3 |  | 1 | 1 | 1 |  |  |  |  |  |  |  |  |  |  |  | 7 |
| A12 |  |  | 2 |  |  |  |  |  |  |  |  |  |  |  |  |  |  |  |  | 2 |
| A13 |  |  | 1 |  |  |  |  |  |  |  |  |  |  |  |  |  |  |  |  | 1 |
| A14 |  |  | 1 |  |  |  |  |  |  |  |  |  |  |  |  |  |  |  |  | 1 |
| A15 |  |  | 2 |  |  |  |  |  |  |  |  |  |  |  |  |  |  |  |  | 2 |
| A16 |  |  | 1 |  |  |  |  |  |  |  |  |  |  |  |  |  |  |  |  | 1 |
| A17 |  |  | 1 |  |  |  |  |  |  |  |  |  |  |  |  |  |  |  |  | 1 |
| A18 |  |  | 1 |  |  |  |  |  |  |  |  |  |  |  |  |  |  |  |  | 1 |
| A19 |  |  | 1 |  |  |  |  |  |  |  |  |  |  |  |  |  |  |  |  | 1 |
| A20 |  |  | 1 |  |  |  |  |  |  |  |  |  |  |  |  |  |  |  |  | 1 |
| A21 |  |  | 1 |  |  |  |  |  |  |  |  |  |  |  |  |  |  |  |  | 1 |
| A22 |  |  |  | 1 |  |  |  |  |  |  |  |  |  |  |  |  |  |  |  | 1 |
| A23 |  |  |  | 1 |  |  |  |  |  |  |  |  |  |  |  |  |  |  |  | 1 |
| A24 |  |  |  | 1 |  |  |  |  |  |  |  |  |  |  |  |  |  |  |  | 1 |
| A25 |  |  |  |  |  |  |  | 1 |  |  |  |  |  |  |  |  |  |  |  | 1 |
| A26 |  |  |  |  |  |  |  | 1 |  |  |  |  |  |  |  |  |  |  |  | 1 |
| A27 |  |  |  |  |  |  | 1 | 1 |  |  |  |  |  |  |  |  |  |  |  | 2 |
| A28 |  |  |  |  |  |  |  | 1 |  |  |  |  |  |  |  |  |  |  |  | 1 |
| A29 |  |  |  |  |  | 1 |  |  |  |  |  |  |  |  |  |  |  |  |  | 1 |
| A30 |  |  |  |  |  | 1 |  |  |  |  |  |  |  |  |  |  |  |  |  | 1 |
| A31 |  |  |  |  |  | 1 |  |  |  |  |  |  |  |  |  |  |  |  |  | 1 |
| A32 |  |  |  |  |  | 1 |  |  |  |  |  |  |  |  |  |  |  |  |  | 1 |
| A33 |  |  |  |  |  | 1 |  |  |  |  |  |  |  |  |  |  |  |  |  | 1 |
| A34 |  |  |  |  |  | 1 |  |  |  |  |  |  |  |  |  |  |  |  |  | 1 |
| A35 |  |  |  |  |  | 1 |  |  |  |  |  |  |  |  |  |  |  |  |  | 1 |
| A36 |  |  |  |  |  |  | 1 |  |  |  |  |  |  |  |  |  |  |  |  | 1 |
| A37 |  |  |  |  |  |  | 1 |  |  |  |  |  |  |  |  |  |  |  |  | 1 |
| A38 |  |  |  |  |  |  | 1 |  |  |  |  |  |  |  |  |  |  |  |  | 1 |
| A39 |  |  |  |  |  |  | 1 |  |  |  |  |  |  |  |  |  |  |  |  | 1 |
| A40 |  |  |  |  |  |  | 1 |  |  |  |  |  |  |  |  |  |  |  |  | 1 |
| A41 |  |  |  |  |  |  |  |  |  |  |  |  | 1 |  |  |  |  |  |  | 1 |
| A42 |  |  |  |  |  |  |  |  |  |  |  |  |  | 1 |  |  |  |  |  | 1 |
| A43 |  |  |  |  |  |  |  |  |  |  |  |  |  | 1 |  |  |  |  |  | 1 |
| A44 |  |  |  |  |  |  |  |  |  | 1 |  |  |  | 1 |  |  |  |  |  | 2 |
| A45 |  |  |  |  |  |  |  |  |  |  |  |  |  | 1 |  |  |  |  |  | 1 |
| A46 |  |  |  |  |  |  |  |  |  |  |  |  |  | 1 |  |  |  |  |  | 1 |
| A47 |  |  |  |  |  |  |  |  |  |  |  |  |  |  | 1 |  |  |  |  | 1 |
| B1 |  |  |  |  |  | 1 |  |  | 1 |  |  |  |  | 1 |  |  |  |  |  | 3 |
| B2 |  |  |  |  |  | 3 |  |  |  |  | 5 |  | 5 | 1 |  |  |  |  |  | 14 |
| B3 |  |  |  |  |  | 1 |  |  |  |  |  |  |  |  |  |  |  |  |  | 1 |
| B4 |  |  |  |  |  |  | 1 |  | 6 |  | 1 |  |  | 2 |  |  |  |  |  | 10 |
| **Haplotype** |  | **South Korea** | | | | | | | | | |  | **Japan** | | | |  | **China** |  | **Total** |
|  |  | **DD** | **UL** | **PH** | **BS** | **TY** | **GJ** | **YS** | **WD** | **SA** | **JJ** |  | **TS** | **TT** | **MY** | **EH** |  | **ZJ** |  |  |
| B5 |  |  |  |  |  |  | 1 |  |  |  |  |  |  |  |  |  |  |  |  | 1 |
| B6 |  |  |  |  |  |  | 1 |  |  |  |  |  | 2 | 2 |  |  |  |  |  | 5 |
| B7 |  |  |  |  |  |  | 1 |  |  |  |  |  |  |  |  |  |  |  |  | 1 |
| B8 |  |  |  |  |  |  |  |  |  |  |  |  | 1 |  |  |  |  |  |  | 1 |
| B9 |  |  |  |  |  |  |  |  |  |  |  |  | 1 |  |  |  |  |  |  | 1 |
| B10 |  |  |  |  |  |  |  |  |  |  |  |  | 1 |  |  |  |  |  |  | 1 |
| B11 |  |  |  |  |  |  |  |  |  |  |  |  | 1 |  |  |  |  |  |  | 1 |
| B12 |  |  |  |  |  |  |  |  |  |  |  |  | 1 |  |  |  |  |  |  | 1 |
| B13 |  |  |  |  |  |  |  |  |  |  |  |  | 1 |  |  |  |  |  |  | 1 |
| B14 |  |  |  |  |  |  |  |  |  |  |  |  | 1 |  |  |  |  |  |  | 1 |
| B15 |  |  |  |  |  |  |  |  |  |  |  |  | 1 |  |  |  |  |  |  | 1 |
| B16 |  |  |  |  |  |  |  |  |  |  |  |  | 1 |  |  |  |  |  |  | 1 |
| B17 |  |  |  |  |  |  |  |  |  |  |  |  | 1 |  |  |  |  |  |  | 1 |
| B18 |  |  |  |  |  |  |  |  |  |  |  |  | 1 |  |  |  |  |  |  | 1 |
| B19 |  |  |  |  |  |  |  |  |  |  |  |  | 1 |  |  |  |  |  |  | 1 |
| B20 |  |  |  |  |  |  |  |  |  |  |  |  | 1 |  |  |  |  |  |  | 1 |
| B21 |  |  |  |  |  |  |  |  |  |  |  |  | 1 |  |  |  |  |  |  | 1 |
| B22 |  |  |  |  |  |  |  |  |  |  |  |  | 1 |  |  |  |  |  |  | 1 |
| B23 |  |  |  |  |  |  |  |  |  |  |  |  | 1 |  |  |  |  |  |  | 1 |
| B24 |  |  |  |  |  |  |  |  |  |  |  |  | 1 |  |  |  |  |  |  | 1 |
| B25 |  |  |  |  |  |  |  |  |  |  |  |  |  | 2 |  |  |  |  |  | 2 |
| B26 |  |  |  |  |  |  |  |  |  |  |  |  |  | 1 |  |  |  |  |  | 1 |
| B27 |  |  |  |  |  |  |  |  |  |  |  |  |  | 1 |  |  |  |  |  | 1 |
| B28 |  |  |  |  |  |  |  |  |  |  |  |  |  | 1 |  |  |  |  |  | 1 |
| B29 |  |  |  |  |  |  |  |  |  |  |  |  |  | 1 |  |  |  |  |  | 1 |
| B30 |  |  |  |  |  |  |  |  |  |  |  |  |  |  |  | 1 |  |  |  | 1 |
| C1 |  |  |  |  |  |  |  |  |  |  |  |  |  |  |  |  |  | 14 |  | 14 |
| C2 |  |  |  |  |  |  |  |  |  |  |  |  |  |  |  |  |  | 9 |  | 9 |
| C3 |  |  |  |  |  |  |  |  |  |  |  |  |  |  |  |  |  | 1 |  | 1 |
| C4 |  |  |  |  |  |  |  |  |  |  |  |  |  |  |  |  |  | 1 |  | 1 |
| C5 |  |  |  |  |  |  |  |  |  |  |  |  |  |  |  |  |  | 15 |  | 15 |
| C6 |  |  |  |  |  |  |  |  |  |  |  |  |  |  |  |  |  | 3 |  | 3 |
| C7 |  |  |  |  |  |  |  |  |  |  |  |  |  |  |  |  |  | 8 |  | 8 |
| C8 |  |  |  |  |  |  |  |  |  |  |  |  |  |  |  |  |  | 1 |  | 1 |
| C9 |  |  |  |  |  |  |  |  |  |  |  |  |  |  |  |  |  | 1 |  | 1 |
| C10 |  |  |  |  |  |  |  |  |  |  |  |  |  |  |  |  |  | 1 |  | 1 |
| C11 |  |  |  |  |  |  |  |  |  |  |  |  |  |  |  |  |  | 4 |  | 4 |
| C12 |  |  |  |  |  |  |  |  |  |  |  |  |  |  |  |  |  | 6 |  | 6 |
| C13 |  |  |  |  |  |  |  |  |  |  |  |  |  |  |  |  |  | 1 |  | 1 |
| C14 |  |  |  |  |  |  |  |  |  |  |  |  |  |  |  |  |  | 1 |  | 1 |
| C15 |  |  |  |  |  |  |  |  |  |  |  |  |  |  |  |  |  | 1 |  | 1 |
| C16 |  |  |  |  |  |  |  |  |  |  |  |  |  |  |  |  |  | 2 |  | 2 |
| C17 |  |  |  |  |  |  |  |  |  |  |  |  |  |  |  |  |  | 1 |  | 1 |
| C18 |  |  |  |  |  |  |  |  |  |  |  |  |  |  |  |  |  | 1 |  | 1 |
| C19 |  |  |  |  |  |  |  |  |  |  |  |  |  |  |  |  |  | 1 |  | 1 |
| C20 |  |  |  |  |  |  |  |  |  |  |  |  |  |  |  |  |  | 1 |  | 1 |
| C21 |  |  |  |  |  |  |  |  |  |  |  |  |  |  |  |  |  | 44 |  | 44 |
| C22 |  |  |  |  |  |  |  |  |  |  |  |  |  |  |  |  |  | 1 |  | 1 |
| C23 |  |  |  |  |  |  |  |  |  |  |  |  |  |  |  |  |  | 1 |  | 1 |
| C24 |  |  |  |  |  |  |  |  |  |  |  |  |  |  |  |  |  | 1 |  | 1 |
| C25 |  |  |  |  |  |  |  |  |  |  |  |  |  |  |  |  |  | 1 |  | 1 |
| C26 |  |  |  |  |  |  |  |  |  |  |  |  |  |  |  |  |  | 1 |  | 1 |
| C27 |  |  |  |  |  |  |  |  |  |  |  |  |  |  |  |  |  | 1 |  | 1 |
| C28 |  |  |  |  |  |  |  |  |  |  |  |  |  |  |  |  |  | 1 |  | 1 |
| C29 |  |  |  |  |  |  |  |  |  |  |  |  |  |  |  |  |  | 1 |  | 1 |
| Total |  | 23 | 104 | 35 | 16 | 31 | 28 | 14 | 12 | 10 | 14 |  | 25 | 30 | 1 | 1 |  | 125 |  | 469 |

**Table S4.** Polymorphic sites of the *COI* haplotype sequence alignment of the three genetic lineages for *Liolophura japonica* inhabiting the northwestern Pacific: Lineage N (A1 − A47), Lineage S1 (B1 – B30), and Lineage S2 (C1 − C29).


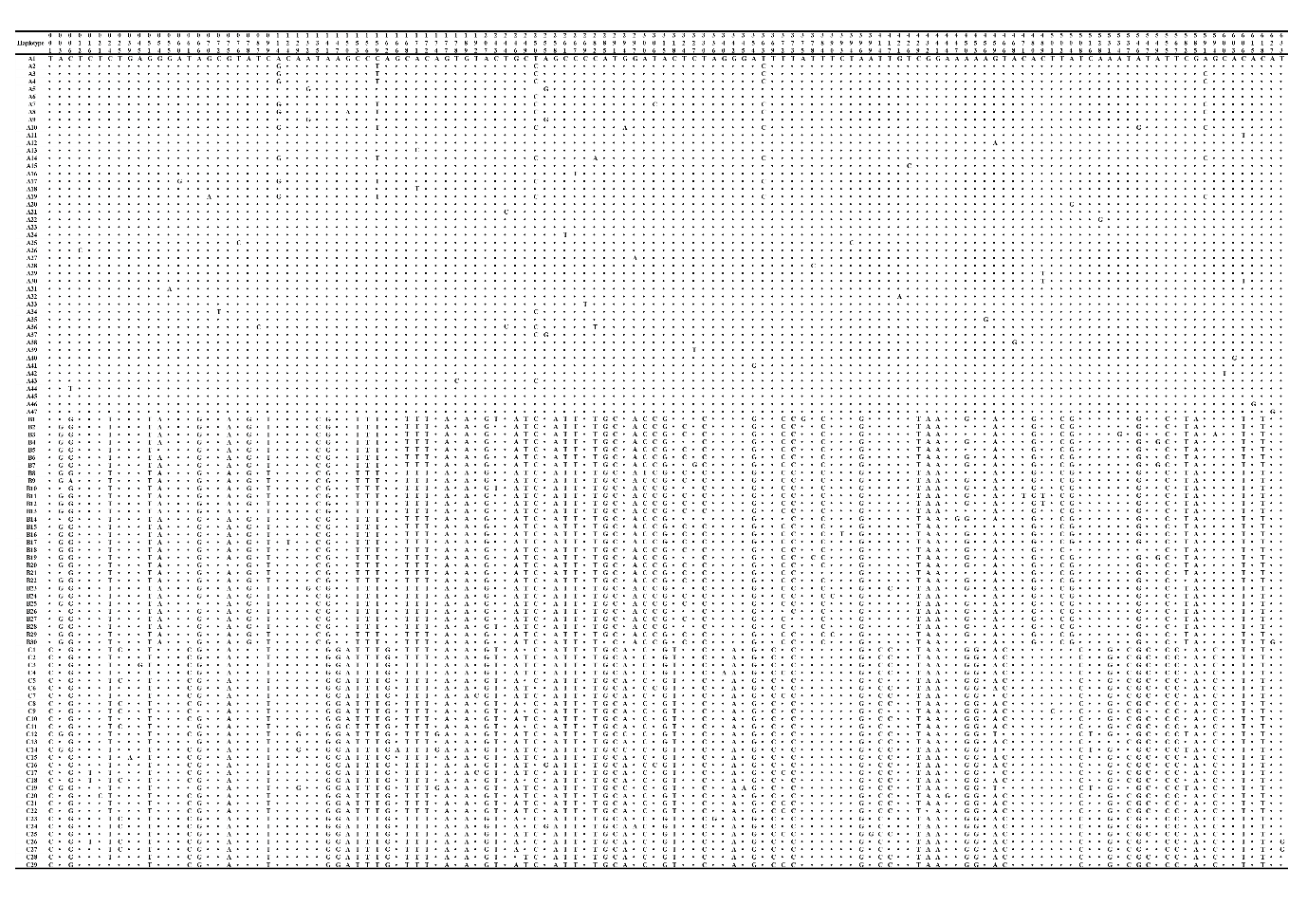
Digits at the top of the figure indicate nucleotide positions. Dots (․) represent the same bases with those of the first-line haplotype A1.

**Table S5.** Summary information of 34 *16S rRNA* haplotypes observed from 425 individuals of *Liolophura japonica* inhabiting the northwestern Pacific.

| **Country** | **Sampling location** | **Latitude** | ***N*** | ***N*_H_** | ***h*** | ***π*** | **References** |
| --- | --- | --- | --- | --- | --- | --- | --- |
| **South Korea** | **Ulleungdo (UL)** | 37.46°N | 104 | 9 | 0.217 | 0.00046 | Present study |
|  | **Dokdo (DD)** | 37.24°N | 23 | 6 | 0.458 | 0.00103 | Present study |
|  | **Pohang (PH)** | 36.02°N | 35 | 3 | 0.113 | 0.00023 | Present study |
|  | **Busan (BS)** | 35.10°N | 16 | 3 | 0.242 | 0.00050 | Present study |
|  | **Tongyeong (TY)** | 34.81°N | 31 | 5 | 0.343 | 0.01162 | Present study |
|  | **Yeosu (YS)** | 34.72°N | 14 | 2 | 0.143 | 0.00029 | Present study |
|  | **Geojedo (GJ)** | 34.70°N | 4 | 3 | 0.833 | 0.00235 | Present study |
|  | **Wando (WD)** | 34.30°N | 12 | 3 | 0.621 | 0.02244 | Present study |
|  | **Sinan (SD)** | 34.34°N | 10 | 1 | 0.000 | 0.00000 | Present study |
|  | **Jeju (JJ)** | 33.24°N | 14 | 3 | 0.582 | 0.02151 | Present study |
| **Japan** | **Tottori(TT)** | 35.53°N | 12 | 2 | 0.303 | 0.00061 | Present study |
|  | **Tsushima(TS)** | 34.45°N | 24 | 5 | 0.312 | 0.00084 | Present study |
| **China** | **Zhejinag (ZJ)** | 27.02–28.00°N | 126 | 11 | 0.662 | 0.00175 | Wu et al. 2018  Retrieved |
| **Total** | | | **425** | **34** | **0.702** | **0.02093** |  |
| ***N***: number of sampled specimens; ***N*_H_**: number of observed haplotypes; ***h***: haplotype diversity; ***π***: nucleotide diversity; n. s.: not significant | | | | | | | |

**Table S6.** Summary of sampling locations, genetic diversity indices and references for *16S rRNA* haplotypes of *Liolophura japonica* inhabiting the northwestern Pacific.

| **No.** | **Species** | **Country** | **Location** | **Accession No.** | **Haplotype** | **Reference** |
| --- | --- | --- | --- | --- | --- | --- |
| **Ingroup** | | | | | | |
| 01 | *Liolophura japonica* | South Korea | Dokdo/Ulleungdo/Pohang Busan/ Tongyeong/Yeosu Wando/Sinan/Jejudo | KT932913 | RA1 | Present study |
| 02 | *Liolophura japonica* | South Korea | Dokdo/Busan | KT932914 | RA2 | Present study |
| 03 | *Liolophura japonica* | South Korea | Dokdo/Ulleungdo, | KT932915 | RA3 | Present study |
| 04 | *Liolophura japonica* | South Korea | Dokdo/Ulleungdo | KT932916 | RA4 | Present study |
| 05 | *Liolophura japonica* | South Korea | Dokdo/Busan | KT932917 | RA5 | Present study |
| 06 | *Liolophura japonica* | South Korea | Dokdo/Ulleungdo, | KT932918 | RA6 | Present study |
| 07 | *Liolophura japonica* | South Korea | Ulleungdo | KT932919 | RA7 | Present study |
| 08 | *Liolophura japonica* | South Korea | Ulleungdo | KT932920 | RA8 | Present study |
| 09 | *Liolophura japonica* | South Korea | Ulleungdo | KT932921 | RA9 | Present study |
| 10 | *Liolophura japonica* | South Korea | Ulleungdo | KT932922 | RA10 | Present study |
| 11 | *Liolophura japonica* | South Korea | Ulleungdo | KT932923 | RA11 | Present study |
| 12 | *Liolophura japonica* | South Korea | Pohang | KT932924 | RA12 | Present study |
| 13 | *Liolophura japonica* | South Korea | Pohang | KT932925 | RA13 | Present study |
| 14 | *Liolophura japonica* | South Korea | Yeosu | KT932926 | RA14 | Present study |
| 15 | *Liolophura japonica* | South Korea | Tongyeong | KT932927 | RA15 | Present study |
| 16 | *Liolophura japonica* | South Korea | Tongyeong | KT932928 | RA16 | Present study |
| 17 | *Liolophura japonica* | South Korea | Tongyeong | KT932929 | RA17 | Present study |
| 18 | *Liolophura japonica* | South Korea Japan | Tongyeong/Geojedo/Wando/Jeju Tsushima/Totori/Amakusa | KT932930  AY377606 | RB1 | Present study  Okusu et al., 2003 |
| 19 | *Liolophura japonica* | South Korea Japan | Geojedo  Tsushima | KT932931 | RB2 | Present study |
| 20 | *Liolophura japonica* | South Korea Japan | Wando/Jeju  Totori | KT932932 | RB3 | Present study |
| 21 | *Liolophura japonica* | Japan | Tsushima | KT932933 | RB4 | Present study |
| 22 | *Liolophura japonica* | Japan | Tsushima | KT932934 | RB5 | Present study |
| 23 | *Liolophura japonica* | Japan | Tsushima | KT932935 | RB6 | Present study |
| 24 | *Liolophura japonica* | China | Zhejiang  unknown | MF716579  MG242630 | RC1 | Wu et al., 2018 |
| 25 | *Liolophura japonica* | China | Zhejiang | MF774368 | RC2 | Wu et al., 2018 |
| 26 | *Liolophura japonica* | China | Zhejiang | MF774369 | RC3 | Wu et al., 2018 |
| 27 | *Liolophura japonica* | China | Zhejiang | MF774370 | RC4 | Wu et al., 2018 |
| 28 | *Liolophura japonica* | China | Zhejiang | MF774371 | RC5 | Wu et al., 2018 |
| 29 | *Liolophura japonica* | China | Zhejiang | MF774372 | RC6 | Wu et al., 2018 |
| 30 | *Liolophura japonica* | China | Zhejiang | MF774373 | RC7 | Wu et al., 2018 |
| 31 | *Liolophura japonica* | China | Zhejiang | MF774374 | RC8 | Wu et al., 2018 |
| 32 | *Liolophura japonica* | China | Zhejiang | MF774375 | RC9 | Wu et al., 2018 |
| 33 | *Liolophura japonica* | China | Zhejiang | MF774376 | RC10 | Wu et al., 2018 |
| 34 | *Liolophura japonica* | China | Zhejiang | MG242644 | RC11 | Unpublished |
| **Outgroup** | | | | | | |
| 35 | *Acanthopleura echinata* | Chile | Valparaiso | MN864062 | - | Irisarri et al., 2020 |

**Table S7.** Geographical distribution along populations of 34 *16S rRNA* haplotypes observed from 425 individuals of *Liolophura japonica* inhabiting the northwestern Pacific.

| **Haplotype** |  | **South Korea** | | | | | | | | | |  | **Japan** | |  | **China** |  | **Total** | |
| --- | --- | --- | --- | --- | --- | --- | --- | --- | --- | --- | --- | --- | --- | --- | --- | --- | --- | --- | --- |
|  |  | **DD** | **UL** | **PH** | **BS** | **TY** | **GJ** | **YS** | **WD** | **SA** | **JJ** |  | **TS** | **TT** |  | **ZJ** |  |  |  |
| RA1 |  | 17 | 92 | 33 | 14 | 23 |  | 13 | 5 | 10 | 8 |  |  |  |  |  |  | 215 |  |
| RA2 |  | 2 |  |  | 1 |  |  |  |  |  |  |  |  |  |  |  |  | 3 |  |
| RA3 |  | 1 | 1 |  |  |  |  |  |  |  |  |  |  |  |  |  |  | 2 |  |
| RA4 |  | 1 | 5 |  |  |  |  |  |  |  |  |  |  |  |  |  |  | 6 |  |
| RA5 |  | 1 |  |  | 1 |  |  |  |  |  |  |  |  |  |  |  |  | 2 |  |
| RA6 |  | 1 | 1 |  |  |  |  |  |  |  |  |  |  |  |  |  |  | 2 |  |
| RA7 |  |  | 1 |  |  |  |  |  |  |  |  |  |  |  |  |  |  | 1 |  |
| RA8 |  |  | 1 |  |  |  |  |  |  |  |  |  |  |  |  |  |  | 1 |  |
| RA9 |  |  | 1 |  |  |  |  |  |  |  |  |  |  |  |  |  |  | 1 |  |
| RA10 |  |  | 1 |  |  |  |  |  |  |  |  |  |  |  |  |  |  | 1 |  |
| RA11 |  |  | 1 |  |  |  |  |  |  |  |  |  |  |  |  |  |  | 1 |  |
| RA12 |  |  |  | 1 |  |  |  |  |  |  |  |  |  |  |  |  |  | 1 |  |
| RA13 |  |  |  | 1 |  |  |  |  |  |  |  |  |  |  |  |  |  | 1 |  |
| RA14 |  |  |  |  |  |  |  | 1 |  |  |  |  |  |  |  |  |  | 1 |  |
| RA15 |  |  |  |  |  | 1 |  |  |  |  |  |  |  |  |  |  |  | 1 |  |
| RA16 |  |  |  |  |  | 1 |  |  |  |  |  |  |  |  |  |  |  | 1 |  |
| RA17 |  |  |  |  |  | 1 |  |  |  |  |  |  |  |  |  |  |  | 1 |  |
| RB1 |  |  |  |  |  | 5 | 2 |  | 1 |  | 5 |  | 20 | 10 |  |  |  | 43 |  |
| RB2 |  |  |  |  |  |  | 1 |  |  |  |  |  | 1 |  |  |  |  | 2 |  |
| RB3 |  |  |  |  |  |  | 1 |  | 6 |  | 1 |  |  | 2 |  |  |  | 10 |  |
| RB4 |  |  |  |  |  |  |  |  |  |  |  |  | 1 |  |  |  |  | 1 |  |
| RB5 |  |  |  |  |  |  |  |  |  |  |  |  | 1 |  |  |  |  | 1 |  |
| RB6 |  |  |  |  |  |  |  |  |  |  |  |  | 1 |  |  |  |  | 1 |  |
| RC1 |  |  |  |  |  |  |  |  |  |  |  |  |  |  |  | 60 |  | 60 |  |
| RC2 |  |  |  |  |  |  |  |  |  |  |  |  |  |  |  | 39 |  | 39 |  |
| RC3 |  |  |  |  |  |  |  |  |  |  |  |  |  |  |  | 12 |  | 12 |  |
| RC4 |  |  |  |  |  |  |  |  |  |  |  |  |  |  |  | 1 |  | 1 |  |
| RC5 |  |  |  |  |  |  |  |  |  |  |  |  |  |  |  | 1 |  | 1 |  |
| RC6 |  |  |  |  |  |  |  |  |  |  |  |  |  |  |  | 7 |  | 7 |  |
| RC7 |  |  |  |  |  |  |  |  |  |  |  |  |  |  |  | 1 |  | 1 |  |
| RC8 |  |  |  |  |  |  |  |  |  |  |  |  |  |  |  | 2 |  | 2 |  |
| RC9 |  |  |  |  |  |  |  |  |  |  |  |  |  |  |  | 1 |  | 1 |  |
| RC10 |  |  |  |  |  |  |  |  |  |  |  |  |  |  |  | 1 |  | 1 |  |
| RC11 |  |  |  |  |  |  |  |  |  |  |  |  |  |  |  | 1 |  | 1 |  |
| Total |  | 23 | 104 | 35 | 16 | 31 | 4 | 14 | 12 | 10 | 14 |  | 24 | 12 |  | 126 |  | 425 |  |

**Table S8.** Polymorphic sites of the *16S rRNA* haplotype sequence alignment of the three genetic lineages for *Liolophura japonica* inhabiting the northwestern Pacific: Lineage N (RA1 –RA17), Lineage S1 (RB1 – RB6), and Lineage S2 (RC1 – RC11).

| Haplotype | 0 | 0 | 0 | 0 | 0 | 0 | 1 | 1 | 1 | 1 | 1 | 2 | 2 | 2 | 2 | 2 | 2 | 2 | 2 | 2 | 2 | 2 | 2 | 2 | 3 | 3 | 3 | 3 | 3 | 3 | 3 | 3 | 3 | 3 | 3 | 3 | 4 | 4 | 4 | 4 | 4 |
| --- | --- | --- | --- | --- | --- | --- | --- | --- | --- | --- | --- | --- | --- | --- | --- | --- | --- | --- | --- | --- | --- | --- | --- | --- | --- | --- | --- | --- | --- | --- | --- | --- | --- | --- | --- | --- | --- | --- | --- | --- | --- |
|  | 0 | 1 | 2 | 4 | 4 | 9 | 3 | 4 | 7 | 8 | 9 | 2 | 3 | 3 | 3 | 4 | 4 | 4 | 5 | 6 | 6 | 6 | 7 | 9 | 0 | 0 | 2 | 2 | 4 | 4 | 4 | 4 | 5 | 5 | 6 | 7 | 0 | 2 | 5 | 6 | 7 |
|  | 6 | 2 | 3 | 4 | 8 | 7 | 9 | 8 | 2 | 9 | 1 | 1 | 0 | 3 | 7 | 4 | 7 | 8 | 2 | 1 | 3 | 7 | 3 | 3 | 4 | 8 | 5 | 6 | 0 | 2 | 4 | 5 | 2 | 3 | 6 | 3 | 4 | 5 | 5 | 9 | 8 |
| RA1 | C | A | G | A | G | C | T | C | T | A | T | A | C | C | G | C | A | C | T | A | A | T | G | A | A | G | A | A | A | A | A | G | A | G | A | A | C | G | A | G | A |
| RA2 | • | • | • | • | • | • | • | • | • | • | • | • | • | • | • | • | G | • | • | • | • | • | • | • | • | • | • | • | • | • | • | • | • | • | • | • | • | • | • | • | • |
| RA3 | • | • | • | • | • | • | • | • | • | • | • | • | • | • | • | • | • | • | • | • | • | • | • | • | • | • | • | • | • | • | • | • | • | • | • | • | • | • | G | • | • |
| RA4 | • | • | • | • | • | • | • | T | • | • | • | • | • | • | • | • | • | • | • | • | • | • | • | • | • | • | • | • | • | • | • | • | • | • | • | • | • | • | • | • | • |
| RA5 | • | • | • | • | • | • | • | • | • | • | • | • | • | • | • | • | • | • | • | • | • | • | • | • | • | • | • | • | • | • | • | • | • | • | • | • | • | • | • | A | • |
| RA6 | • | • | • | • | • | T | • | • | • | • | • | • | • | • | • | • | • | • | • | • | • | • | • | • | • | • | • | • | • | • | • | • | • | • | • | • | • | • | • | • | • |
| RA7 | • | • | • | • | • | • | • | • | • | • | • | • | • | • | • | • | • | • | • | • | • | • | • | • | • | • | • | • | • | • | • | • | • | • | • | • | • | • | • | • | • |
| RA8 | • | • | • | • | • | • | • | • | • | • | • | • | • | • | • | • | • | • | • | • | • | • | • | • | • | • | • | • | • | • | G | • | • | • | • | • | • | • | • | • | • |
| RA9 | • | • | • | • | • | • | • | • | • | • | • | • | • | • | • | • | • | T | • | • | • | • | • | • | • | • | • | • | • | • | • | • | • | • | • | • | • | • | • | • | • |
| RA10 | • | • | • | C | • | • | • | • | • | • | • | • | • | • | • | • | • | • | • | • | • | • | • | • | • | • | • | • | • | • | • | • | • | • | • | • | • | • | • | • | • |
| RA11 | • | • | • | • | • | • | • | • | • | • | C | • | • | • | • | • | • | • | • | • | • | • | • | • | • | • | • | • | • | • | • | • | • | • | • | • | • | • | • | • | • |
| RA12 | • | • | • | • | • | • | • | • | • | • | • | G | • | • | • | • | • | • | • | • | • | • | • | • | • | • | • | • | • | • | • | • | • | • | • | • | • | • | • | • | • |
| RA13 | • | • | • | • | • | • | • | • | • | • | • | • | • | • | A | • | • | • | • | • | • | • | • | • | • | • | • | • | • | • | • | • | • | • | • | • | • | • | • | • | • |
| RA14 | • | • | • | • | • | • | • | • | • | • | • | • | • | • | • | • | • | • | • | • | • | • | • | • | • | • | • | • | • | G | • | • | • | • | • | • | • | • | • | • | • |
| RA15 | • | • | • | • | • | • | • | • | • | • | • | • | • | • | • | • | • | • | • | • | • | • | • | • | • | • | • | • | • | • | • | A | • | • | • | • | • | • | • | • | • |
| RA16 | • | • | • | • | • | • | • | • | • | • | • | • | • | • | • | • | • | • | • | • | • | • | • | • | • | • | • | • | • | • | • | • | C | • | • | • | • | • | • | • | • |
| RA17 | • | • | • | • | • | • | • | • | • | • | • | • | • | • | • | G | • | • | • | • | • | • | • | • | • | • | • | • | • | • | • | • | • | • | • | • | • | • | • | • | • |
| RB1 | T | • | A | • | A | • | • | A | C | • | • | • | T | T | A | T | G | T | • | • | G | • | A | • | • | A | G | G | G | • | • | • | • | A | G | • | • | A | • | • | • |
| RB2 | T | • | A | • | A | • | • | A | C | G | • | • | T | T | A | T | G | T | C | • | G | • | A | • | • | A | G | G | G | • | • | • | • | A | G | • | • | A | • | • | • |
| RB3 | T | • | A | • | A | • | • | A | C | G | • | • | T | T | A | T | G | T | • | • | G | • | A | • | • | A | G | G | G | • | • | • | • | A | G | • | • | A | • | • | • |
| RB4 | T | • | A | • | A | • | • | A | C | • | • | • | T | T | A | T | G | T | • | • | G | • | A | • | • | A | G | G | G | • | • | • | • | A | G | • | T | A | • | • | • |
| RB5 | T | G | A | • | A | • | • | A | C | • | • | • | T | T | A | T | G | T | • | • | G | • | A | • | • | A | G | G | G | • | • | • | • | A | G | • | • | A | • | • | • |
| RB6 | T | • | A | • | A | • | • | A | C | • | • | • | T | T | A | T | G | T | • | • | G | • | • | • | • | A | G | G | G | • | • | • | • | A | G | • | • | A | • | • | • |
| RC1 | T | • | A | • | A | • | • | A | • | • | • | • | T | T | A | T | G | • | • | • | G | • | A | • | G | A | G | G | G | • | • | • | • | A | G | • | • | A | • | • | G |
| RC2 | T | • | A | • | A | • | • | A | • | • | • | • | T | T | • | T | G | • | • | • | G | • | A | • | G | A | G | G | G | • | • | • | • | A | G | • | • | A | • | • | G |
| RC3 | T | • | A | • | A | • | • | A | • | • | • | • | T | T | A | T | G | • | • | G | G | • | A | • | G | A | G | G | G | • | • | • | • | A | G | • | • | A | • | • | G |
| RC4 | T | • | A | • | A | • | C | A | • | • | • | • | T | T | • | T | G | • | • | • | G | • | A | • | G | A | G | G | G | • | • | • | • | A | G | • | • | A | • | • | G |
| RC5 | T | • | A | • | A | • | • | A | • | • | • | • | T | T | A | T | G | • | • | • | G | • | A | • | G | A | G | G | G | • | • | • | • | A | G | • | • | A | • | A | G |
| RC6 | T | • | A | • | A | • | • | G | • | • | • | • | T | T | A | T | G | • | • | • | G | • | A | • | G | A | G | G | G | • | • | • | • | A | G | • | • | A | • | • | G |
| RC7 | T | • | A | • | A | • | • | A | • | • | • | • | T | • | A | T | G | • | • | • | G | • | A | • | G | A | G | G | G | • | • | • | • | A | G | • | • | A | • | • | G |
| RC8 | T | • | A | • | A | • | • | G | • | • | • | • | T | T | A | T | G | • | • | • | G | C | A | • | G | A | G | G | G | • | • | • | • | A | G | • | • | A | • | • | G |
| RC9 | T | • | A | • | A | • | • | A | • | • | • | • | T | T | • | T | G | • | • | • | G | • | A | G | G | A | G | G | G | • | • | • | • | A | G | • | • | A | • | • | G |
| RC10 | T | • | A | • | A | • | • | A | • | • | • | • | T | T | • | T | G | • | • | • | G | • | A | • | G | A | G | G | G | • | • | • | • | A | G | G | • | A | • | • | G |
| C11 | T | • | A | • | A | • | • | A | • | • | • | • | T | T | A | T | G | • | • | • | G | • | A | • | G | A | G | G | G | • | • | • | • | A | G | • | • | A | • | • | G |

Digits at the top of the figure indicate nucleotide positions. Dots (․) represent the same bases with those of the first-line haplotype RA1.

**Table S9.** The result of the AMOVA test performed with the three *COI* genetic lineages (N, S1, and S2) of *Liolophura japonica*.

| **Grouping** | **Source of Variation** | **Degree of freedom** | **Sum of squares** | **Variance components** | **Percentage of variation** | ***p*** |
| --- | --- | --- | --- | --- | --- | --- |
| One gene pool in the Lineage N | Among populations | 9 | 195.678 | 13.46600 | 90.19 | <0.05 |
|  | Within populations | 117 | 171.463 | 1.46600 | 9.81 |  |
| One gene pool in the Lineage S1 | Among populations | 5 | 80.676 | -13.94228 | 128.01 | >0.05 |
|  | Within populations | 20 | 61.017 | 3.05083 | -28.01 |  |
| One gene pool in the Lineage S2 | Among populations | 12 | 276.550 | 5.39125 | 54.01 | >0.05 |
|  | Within populations | 49 | 224.986 | 4.59156 | 45.99 |  |
| One gene pool in the Lineage S1 & S2 | Among populations | 18 | 2575.929 | 38.86867 | 90.36 | <0.001 |
|  | Within populations | 69 | 286.003 | 4.14497 | 9.64 |  |
| Three lineages  (Lineage N vs. Lineage S1 vs. Lineage S2 ) | Among groups | 2 | 13226.397 | -78.26965 | 103.08 | >0.05 |
|  | Among populations within groups | 26 | 47.718 | -0.12228 | 0.16 | <0.05 |
|  | Within populations | 186 | 457.467 | 2.45950 | -3.24 | <0.001 |
| Two gene pool  (Lineage N vs. Lineage S1 & S2) | Among groups | 1 | 12117.045 | -53.10165 | 122.90 | <0.001 |
|  | Among populations within groups | 27 | 1157.070 | 7.43455 | -17.21 | <0.001 |
|  | Within populations | 186 | 457.467 | 2.45950 | -5.69 | >0.05 |

**Table S10.** The result of the AMOVA test performed with the three *16S rRNA* genetic lineages (N, S1, and S2) of *Liolophura japonica*.

| **Grouping** | **Source of Variation** | **Degree of freedom** | **Sum of squares** | **Variance components** | **Percentage of variation** | ***p*** |
| --- | --- | --- | --- | --- | --- | --- |
| One gene pool in the Lineage N | Among populations | 5 | 24.328 | -0.98023 | 131.24 | >0.05 |
|  | Within populations | 99 | 23.101 | 0.23334 | -31.24 |  |
| One gene pool in the Lineage S1 | Among populations | 4 | 11.272 | -1.64446 | 137.91 | >0.05 |
|  | Within populations | 20 | 9.042 | 0.45208 | -37.91 |  |
| One gene pool in the Lineage S2 | Among populations | 13 | 62.880 | 1.20554 | 55.49 | >0.05 |
|  | Within populations | 49 | 47.390 | 0.96714 | 44.51 |  |
| One gene pool in the Lineage S1 & S2 | Among populations | 18 | 374.936 | 5.59747 | 87.25 | <0.001 |
|  | Within populations | 69 | 56.431 | 0.81785 | 12.75 |  |
| Three lineages  (Lineage N vs. Lineage S1 vs. Lineage S2 ) | Among groups | 2 | 3969.557 | -30.55305 | 101.54 | >0.05 |
|  | Among populations within groups | 22 | 9.474 | -0.00914 | 0.03 | <0.05 |
|  | Within populations | 168 | 79.532 | 0.47340 | -1.57 | <0.001 |
| Two gene pool  (Lineage N vs. Lineage S1 & S2) | Among groups | 1 | 3819.165 | -19.48083 | 109.84 | >0.05 |
|  | Among populations within groups | 23 | 159.866 | 1.27209 | -7.17 | <0.001 |
|  | Within populations | 168 | 79.532 | 0.47340 | -2.67 | <0.001 |

**Table S11.** Summary of the neutrality tests performed with *COI* and *16S rRNA* haplotypes along *Liolophura koreana*, sp. nov. *Liolophura japonica*, and *L*. *sinensis*, sp. nov. inhabiting the northwestern Pacific.

| **Gene** | **Species** | | ***N*_H_^1)^** | **Tajima`s *D*** | **Fu`s *F*s** |
| --- | --- | --- | --- | --- | --- |
| *COI* | *L*. *koreana* | 47 | | **-2.202^*^** | **-26.017^**^** |
|  | *L*. *japonica* | 30 | | **-2.070^*^** | **-25.970^**^** |
|  | *L*. *sinensis* | 29 | | -1.400 | **-25.268^**^** |
| *16S rRNA* | *L*. *koreana* | 17 | | **-2.319^**^** | **-21.775^**^** |
|  | *L*. *japonica* | 6 | | -0.826 | **-4.553^*^** |
|  | *L*. *sinensis* | 11 | | -1.320 | **-11.458^**^** |

^1)^ The detail information of the numbers of haplotypes and employed individuals refers to Supplementary Tables 1, 2, 5, and 6; ^*^: p<0.01, ^**^: p<0.001.

**Data S1.** Nucleotide sequence alignment of 106 *COI* haplotypes of *Liolophura japonica* with an outgroup.

**Data S2.** Nucleotide sequence alignment of 34 *16S rRNA* haplotypes of *Liolophura japonica* with an outgroup.

**Data S3.** Nucleotide sequence alignment of 106 *COI* haplotypes of *Liolophura japonica, Liolophura koreana* sp. nov., and *Liolophura sinensis* sp. nov., one *COI* haplotype of *Liolophura tenuispinosa*, and 14 *COI* haplotypes of eight *Acanthopluera* congeneric species with an outgroup *Tonicia forbesii*.
